# Adipose Tissue‐Resident *Sphingomonas Paucimobilis* Suppresses Adaptive Thermogenesis by Reducing 15‐HETE Production and Inhibiting AMPK Pathway

**DOI:** 10.1002/advs.202310236

**Published:** 2024-10-30

**Authors:** Yucheng Zhu, Ruiqi Yang, Zhangchao Deng, Bohua Deng, Kun Zhao, Chen Dai, Gang Wei, YanJiang Wang, Jinshui Zheng, Zhuqing Ren, Wentao Lv, Yingping Xiao, Zhinan Mei, Tongxing Song

**Affiliations:** ^1^ College of Animal Science and Technology Huazhong Agricultural University Wuhan 430070 China; ^2^ Department of Endocrinology the Seventh Medical Center of Chinese PLA General Hospital Beijing 100700 China; ^3^ Institute of Organ Transplantation Tongji Hospital Tongji Medical College Huazhong University of Science and Technology Wuhan 430030 China; ^4^ Beijing Key Laboratory of Diabetes Research and Care Department of Endocrinology, Beijing Diabetes Institute Beijing Tongren Hospital Capital Medical University Beijing 100730 China; ^5^ Beijing Chao‐yang Hospital Capital Medical University Beijing 100020 China; ^6^ State Key Laboratory of Agricultural Microbiology Huazhong Agricultural University Wuhan 430070 China; ^7^ State Key Laboratory for Managing Biotic and Chemical Threats to the Quality and Safety of Agro‐Products Institute of Agro‐Product Safety and Nutrition Zhejiang Academy of Agricultural Sciences Hangzhou 310021 China; ^8^ College of Plant Science and Technology Huazhong Agricultural University Wuhan 430070 China

**Keywords:** 15‐HETE, adaptive thermogenesis, adipose tissues, AMPK, chronic inflammation, microbes

## Abstract

Obesity represents a low‐grade chronic inflammation status, which is associated with compromised adaptive thermogenesis. However, the mechanisms underlying the defective activation of thermogenesis in chronic inflammation remain unclear. Here, a chronic inflammatory model is first estabolished by injecting mice with low‐dose lipopolysaccharide (LPS) before cold exposure, and then it is verified that LPS treatment can decrease the core body temperature of mice and alter the microbial distribution in epididymal white adipose tissue (eWAT). An adipose tissue‐resident bacterium *Sphingomonas paucimobilis* is identified as a potential inhibitor on the activation of brown fat and browning of inguinal WAT, resulting in defective adaptive thermogenesis. Mechanically, LPS and *S. paucimobilis* inhibit the production and release of 15‐HETE by suppressing its main metabolic enzyme 12 lipoxygenase (12‐LOX) and 15‐ Hydroxyeicosatetraenoic acid (15‐HETE) rescues the impaired thermogenesis. Interestingly, 15‐HETE directly binds to AMP‐activated protein kinase α (AMPKα) and elevates the phosphorylation of AMPK, leading to the activation of uncoupling protein 1 (UCP1) and mitochondrial oxidative phosphorylation (OXPHOS) complexes. Further analysis with human obesity subjects reveals that individuals with high body mass index displayed lower 15‐HETE levels. Taken together, this work improves the understanding of how chronic inflammation impairs adaptive thermogenesis and provides novel targets for alleviating obesity.

## Graphical abstract

Lipopolysaccharide (LPS) induces defective adaptive thermogenesis through an adipose tissue‐resident *S. paucimobilis*–15‐HETE–AMPK–UCP1 axis, providing a new target for activating thermogenesis and thereby ameliorating obesity and the metabolic syndrome.

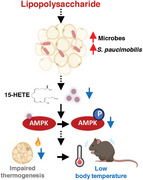

## 1 Introduction

Obesity and associated metabolic complications such as type 2 diabetes (T2D) are becoming increasingly prevalent worldwide, representing a major socio‐economic burden in the last few decades.^[^ ^1^, ^2^ ^]^ There is an urgent need to discover novel therapeutic strategies to treat obesity and its related chronic diseases. Obesity can be indicated by a high body mass index (BMI), which is calculated as body weight in kilograms divided by the squared value of body height in meters (kg m⁻^2^).^[^ ^3^ ^]^ Due to the positive role of brown adipose tissue (BAT) and browning of white adipose tissue (WAT) in energy balance (i.e., energy expenditure known as adaptive thermogenesis), consumption of glucose, and insulin sensitivity, the activation of BAT and selective induction of WAT browning are believed to have therapeutic potential in counteracting the obesity pandemic.^[^ ^4^, ^5^ ^]^ Cold exposure has been found to promote BAT activity, thereby elevating energy expenditure, facilitating weight loss, and ameliorating metabolic disorders.^[^ ^6^ ^]^ However, adaptive thermogenesis is often compromised in obese subjects^[^ ^7^ ^]^ or in mice under lipopolysaccharide (LPS) treatment,^[^ ^8^ ^]^ which largely blunts its effect to combat obesity. Notably, obesity has been identified as a chronic low‐grade inflammation disease characterized by the increase in endotoxins and release of pro‐inflammatory cytokines.^[^ ^9^, ^10^, ^11^ ^]^ The endotoxin LPS is elevated in both the circulation and adipose tissue under obesity,^[^ ^9^, ^12^, ^13^ ^]^ which is accompanied by decreases in the expression of uncoupling protein 1 (UCP1) in adipocytes.^[^ ^8^, ^14^, ^15^ ^]^ The molecular mechanisms responsible for the impaired adaptive thermogenesis in chronic inflammation have not been fully elucidated yet.

Recently, there have been several reports about the presence of bacteria and bacterial DNA in several adipose tissues under obesity and T2D.^[^ ^13^, ^16^, ^17^ ^]^ The presence of adipose tissue‐resident microbes and related signatures in individuals with severe obesity implies defective BAT activity under obesity.^[^ ^16^, ^17^ ^]^ However, the role of tissue‐resident bacteria in adaptive thermogenesis remains unclear.

To fill the gap, we established a compromised adaptive thermogenesis model by LPS administration. LPS administration increased the number and altered the composition of adipose tissue‐resident microbes. One of the key adipose tissue‐resident bacteria, *Sphingomonas paucimobilis*, was mainly distributed in epididymal WAT (eWAT). LPS promoted the translocation of *S. paucimobilis* from the gut to eWAT. Furthermore, gavage of *S. paucimobilis* suppressed the level of UCP1 and mitochondrial oxidative phosphorylation (OXPHOS) complexes in BAT and inguinal WAT (iWAT), leading to abolishment of adaptive thermogenesis by downregulating 15‐ Hydroxyeicosatetraenoic acid (15‐HETE) in a 12‐lipoxygenase (12‐LOX) dependent manner. We also found that 15‐HETE directly binds to AMP‐activated protein kinase α (AMPKα) and elevates AMPK phosphorylation, which augments the expression of downstream thermogenic proteins. These results explain how LPS induces defective adaptive thermogenesis through a *S. paucimobilis*–15‐HETE–AMPK–UCP1 axis, providing a new target for activating thermogenesis and thereby ameliorating obesity and the metabolic syndrome.

## 2 Results

### 2.1 Chronic Inflammation Induced by LPS Increases the Accumulation of Microbes in Visceral Adipose Tissues

**Figure 1:**
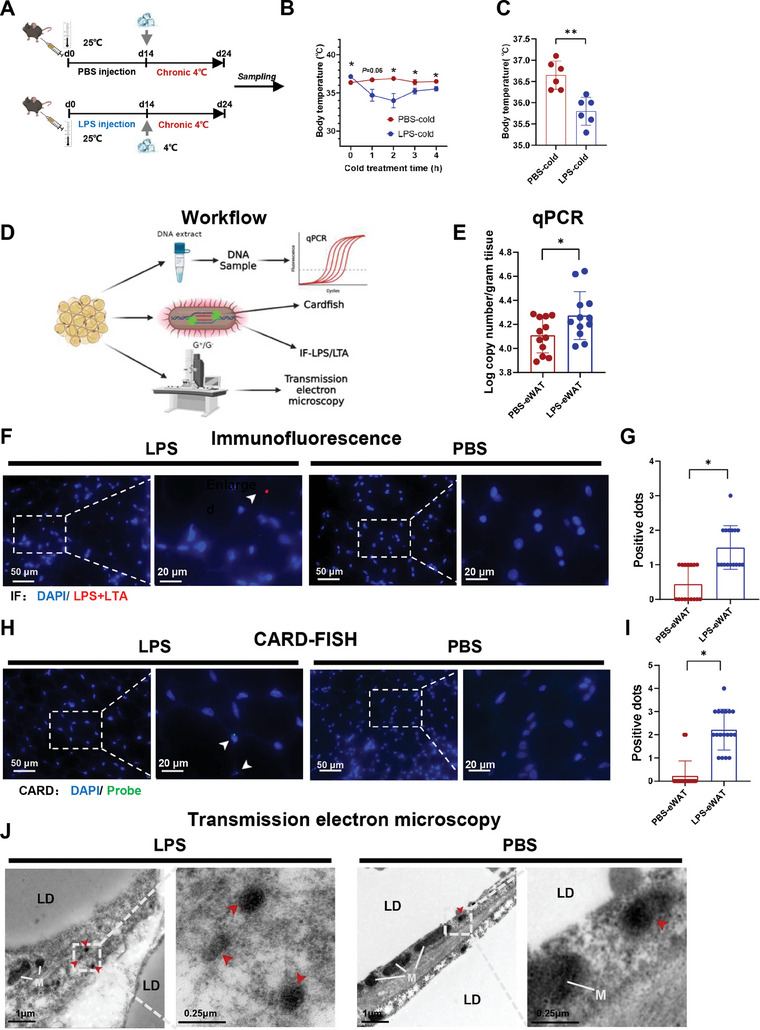
Adipose tissue‐resident microbes in LPS‐induced chronic inflammation during cold exposure. A) Schematic diagram of cold exposure experiment. B) Core body temperature in PBS‐injected mice with cold treatment (PBS‐Cold) and LPS‐injected mice with cold treatment (LPS‐Cold) groups in 4 h after cold treatment, *n* = 6. C) Body temperature changes in PBS‐Cold and LPS‐Cold groups in 10d, *n* = 6. D) Schematic diagram showing the workflow for demonstrating the presence of bacteria in adipose tissues. E) qPCR quantification of the bacteria in eWAT, *n* = 12. F,G) IF staining of LPS+LTA (red) to show the localization of gram‐positive and gram‐negative bacteria in eWAT. LPS+LTA (red) and nuclei (blue). Scale bar is 50 µm, Scale bar is 20 µm for enlarged image, *n* = 16. H,I) CARD‐FISH to show the localization of total bacteria in eWAT. Probe (green) and nuclei (blue), *n* = 18. J) TEM image of eWAT showing bacteria structures within adipose tissues. Red arrows point to bacterial structures. LD, lipid droplet; M, mitochondria; N, nucleus. Scale bar is 1 µm, Scale bar is 0.25 µm for enlarged image. * *p* < 0.05 and ** *p* < 0.01.

Previous research has demonstrated that chronic injection of LPS impairs cold‐induced thermogenesis.^[^ ^8^, ^14^ ^]^ Consistently, we found that two‐week chronic LPS administration by intraperitoneal injection significantly reduced the core body temperature compared with  Phosphate‐buffered saline (PBS)‐treated mice that were exposed to cold for 10 days (**Figure** **1**A–C). LPS‐treated mice exhibited a significant decrease in the browning of iWAT, indicating a negative impact of chronic inflammation on adaptive thermogenesis (Figure S1A, Supporting Information). Since there have been reports about the presence of intratissue microbes in human adipose tissue,^[^ ^16^, ^17^ ^]^ we determined the composition of adipose tissue‐resident microbes to elucidate the underlying mechanism for tissue‐resident microbes to influence adaptive thermogenesis. Real‐time Quantitative PCR (qPCR), Immunofluorescence (IF), Catalyzed reporter deposition fluorescent in situ hybridization (CARD‐FISH), and transmission electron microscopy (TEM) were employed to analyze the resident microbes in adipose tissue samples (Figure 1D). Due to the low biomass of tissue microbes and highly possible contamination caused by DNA in the tissue and environment, we conducted the qPCR through multiple steps and detected a median of 1.0 × 10^4^ bacteria per gram of tissue in PBS‐eWAT, while a nearly twofold bacterial load in LPS‐eWAT (2.0 × 10^4^ bacteria per gram) (Figure 1E). Relatively lower bacterial loads were detected in other kinds of adipose tissue, such as iWAT and BAT (Figure S1B,C, Supporting Information). We further analyzed the distribution characteristics of the microbes in adipose tissues. The immunofluorescence analysis by targeting LPS (for gram‐negative bacteria) and lipoteichoic acid (LTA) (for gram‐positive bacteria) and CARD‐FISH by targeting 16S rRNA gene fragments all showed the presence of bacteria in the perinuclear and cytosol region (Figure 1F–I). To determine the spatial location of microbes in adipose tissues, we further performed tissue clearing and collected data from 3D tissues for statistical analysis. A total of 60 sections were stacked up with a total volume of 371 × 371 × 60 µm^3^. The results revealed that LPS treatment significantly increased the bacterial number in eWAT (Figure S1D,E, Supporting Information). Furthermore, TEM analysis revealed that the majority of bacteria‐like structures were located in the cytosol and extracellular space (Figure 1J). Taken together, these results suggested the presence of microbes in adipose tissue and that LPS treatment increases the number of microbes under cold treatment.

### 2.2 *Sphingomonas* from *α‐Proteobacteria* is Mainly Enriched in eWAT in Response to LPS Treatment

**Figure 2:**
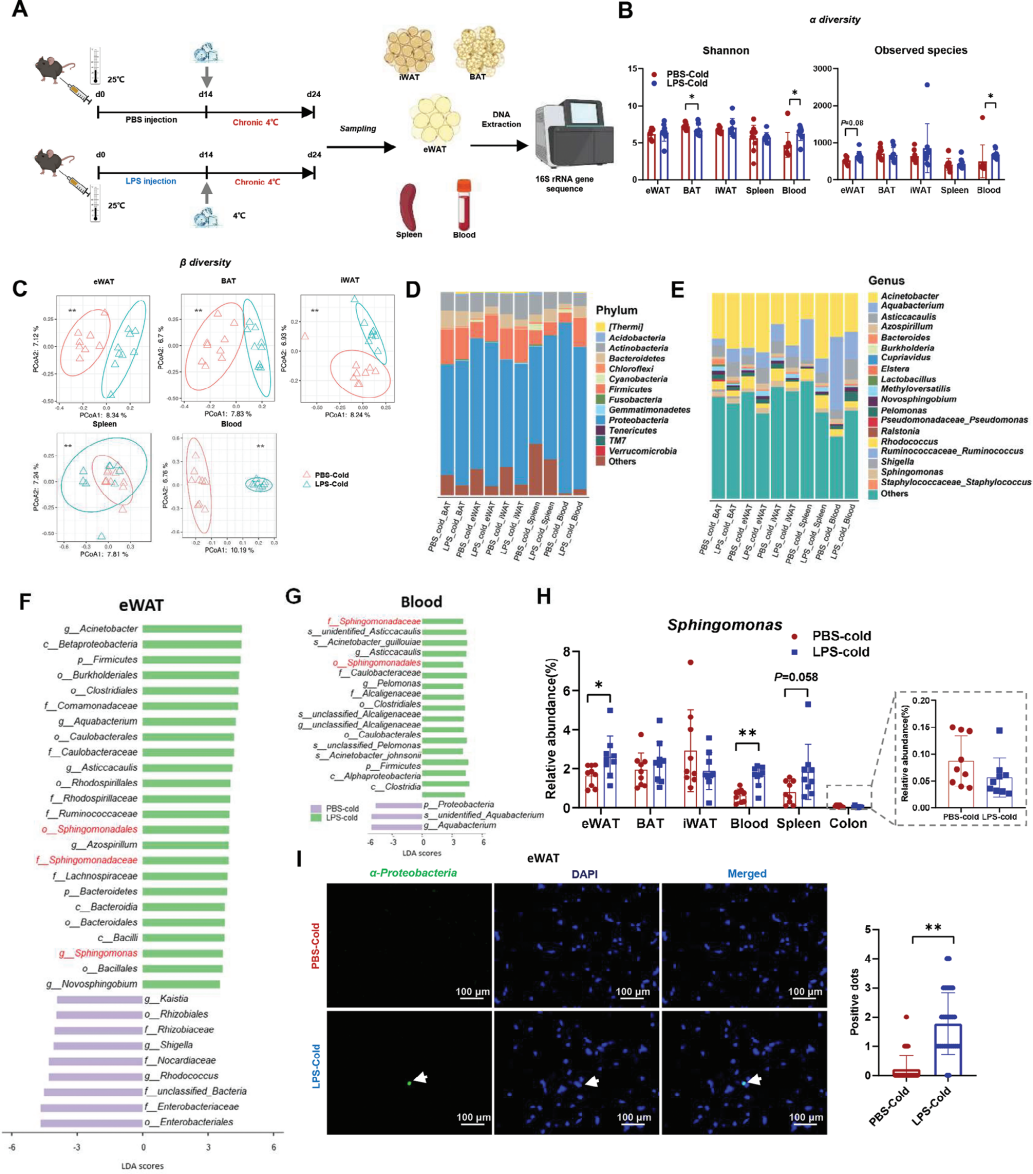
Cold exposure changes the composition of *Sphingomonas*, a tissue‐resident key bacterium responsive to LPS. A) Schematic diagram of cold exposure experiment. B) Shannon index and Observed diversity in adipose tissues, spleen, and blood, *n* = 9. Differences in α‐diversity of each group were tested by Wilcoxon rank‐sum test. *p < 0.05; ** *p* < 0.01. C) Principal coordinate analysis (PCoA) based on Jaccard distance revealed microbiota clustering in different tissues of PBS‐Cold and LPS‐Cold groups. The percentage of variation explained by the plotted principal coordinates is indicated in the axis labels. Differences in β‐diversity in each group were tested by Adonis. * P < 0.05; ** *p* < 0.01. D,E) Relative bacterial abundance at the phylum (D) and genus (E) level in different tissues of PBS‐Cold and LPS‐Cold groups. F,G) Most differential taxa in the microbiota of eWAT (F) and blood (G) between PBS‐Cold and LPS‐Cold groups. LDA Score: LDA score. H) Relative abundance of *Sphingomonas* in different tissues of PBS‐Cold and LPS‐Cold groups, *n* = 9. * *p* < 0.05 and ** *p* < 0.01. I) CARD‐FISH on eWAT sections from PBS‐Cold and LPS‐Cold groups: *α‐Proteobacteria* (green) and nuclei (blue). Scale bar is 100 µm, *n* = 31. Quantitative analysis of positive dots in each group.

To assess the microbial characteristics of adipose tissue after LPS injection under cold treatment, we investigated the composition of adipose tissue‐resident microbes to elucidate the underlying mechanism for the effect of adipose tissue‐resident microbes on adaptive thermogenesis. 16S rRNA gene sequencing was employed to analyze the translocation of microbes among different tissues, including BAT, iWAT, eWAT, whole blood, and spleen. A significant difference in α‐diversity in the blood and eWAT was observed between the LPS and PBS treatment groups, as indicated by the observed species and Shannon indices (**Figure** **2**A,B). The β‐diversity analysis results showed that LPS‐treated mice had a significantly different microbial composition from the PBS‐treated mice (Figure 2C). Moreover, a significant difference was observed between microbes in the colon and those in other tissues (Figure S2A–G, Supporting Information). Taxonomy at the phylum level was predominated by *Proteobacteria* and *Firmicutes* in adipose tissues, which was different from the predominant phyla (*Bacteroidetes* and *Firmicutes*) in gut microbes (Figure 2D; Figure S2E, Supporting Information). At the genus level, there were several remarkable changes in microbial composition, such as *Sphingomonas*, *Pseudomonas*, *Acinetobacter*, and *Aquabacterium* (Figure 2E). Together, the significantly distinct microbial compositions among tissues (Figure S2F,G, Supporting Information) indicated a low risk of contamination from the environment and implied the distinct functions of tissue‐resident microbes.

Given the significance of bacterial composition in blood and eWAT, we investigated which bacterial species are key species under LPS treatment. The linear discriminant analysis (LDA) effect size (LEfSe) method with a log_10_ LDA score > 3.5 demonstrated that the LPS group was characterized by *Sphingomonas*, which belongs to the order *α‐Proteobacteria* (Figure 2F,G). Consistently, the relative abundance of *Sphingomonas* significantly increased in eWAT and blood of LPS treatment group compared with that in PBS treatment group, but was very low in the colon content with no statistically significant difference (Figure 2H), indicating that these tissue‐resident species are probably involved in regulating the target tissues. To characterize the location of the microbes in eWAT, we performed a CARD‐FISH analysis using probes for *α‐Proteobacteria*. More bacterial signals were observed in the eWAT from LPS‐treated mice (Figure 2I). Next, we successfully isolated and identified a species of *Sphingomonas*, namely *Sphingomonas paucimobilis*,^[^ ^18^ ^]^ which will be used for further biological functional validation. Taken together, these results indicated that the specific bacterial distribution in eWAT may be disturbed by LPS treatment and the species belonging to *Sphingomonas* may be a vital candidate that regulates the biological function of eWAT during cold exposure.

### 2.3 LPS Promotes the Translocation of *S. paucimobilis* from the Gut to eWAT

**Figure 3:**
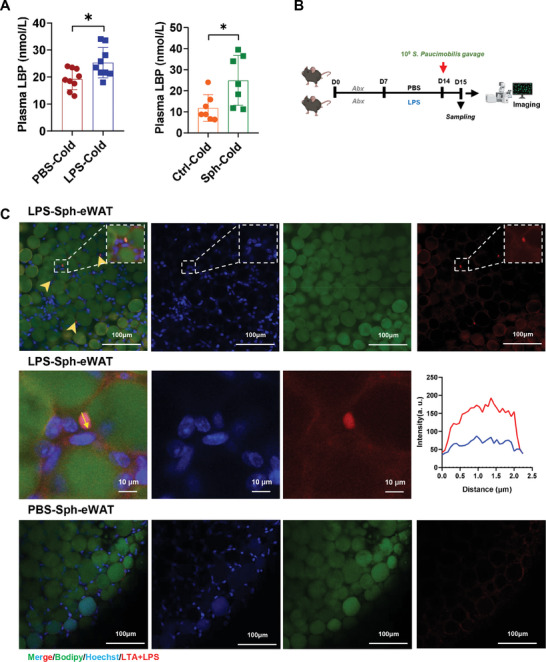
LPS induces *S. paucimobilis* to translocate from the gut to eWAT. A) Serum LBP level in PBS‐Cold and LPS‐Cold groups (*n* = 9), Ctrl‐Cold and *S. paucimobilis* (Sph)p‐Cold groups (*n* = 7). B) Schematic diagram of *S. paucimobilis* tracking experiment in vivo in LPS treated mice. C) Confocal images of eWAT of LPS or PBS injection mice gavaged with *S. paucimobilis* stained with LPS and LTA antibody (red), Hoechst (blue), and Bodipy (green). Relative intensity within the indicated yellow area was measured. Scale bar is 100 µm, Scale bar is 10 µm for enlarged image. *p < 0.05 and **p < 0.01.

Disruption of gut barrier, especially intestinal permeability, has been proposed as a potential mechanism for bacterial translocation.^[^ ^19^ ^]^ To determine whether *S. paucimobilis* originates from the gut microbiota, we measured the serum concentration of lipopolysaccharide‐binding protein (LBP),^[^ ^20^ ^]^ a surrogate marker of intestinal permeability. The results showed that LPS treatment significantly increased the serum level of LBP, which is consistent with the results of *S. paucimobilis* oral gavage (**Figure** **3**A), indicating possible intestinal leak of microbes under chronic inflammation. These results suggested that impairment of intestinal barrier provides a potential way for the dissemination of bacteria into the circulation and distal tissues.

To assess whether LPS enhances the translocation of bacteria into eWAT, antibiotic‐treated mice were injected with LPS, followed by oral gavage of *S. paucimobilis*, and the presence of bacteria was determined by tissue‐clearing and IF. The results showed that LPS treatment increased the bacterial translocation and colonization in eWAT (Figure 3B,C) but few positive dots were observed in iWAT (Figure S3A, Supporting Information). Next, we engineered GFP plasmid‐transformed *Escherichia coli (E. coli)* since GFP failed to be expressed in *S. paucimobilis*, and gavaged mice with the engineered *E. coli* for spatial tracing of the bacteria. The results revealed that GFP‐*E. coli* colonized in eWAT and iWAT after LPS treatment (Figure S3B–E, Supporting Information), suggesting that LPS induces the translocation of bacteria from the gut to eWAT. Then, we labeled live *S. paucimobilis* with a fluorescent D‐amino acid (FDAA)‐based strategy^[^ ^21^ ^]^ and gavaged germ‐free mice with labeled bacteria for 0 h, 6 h, 12 h, and 24 h (Figure S4A,B, Supporting Information). The small intestine (ileum), colon, and eWAT were sampled and 3D imaged after tissue clearing with a confocal microscope (Figure S4C–F, Supporting Information). Labeled *S. paucimobilis* was found in the small intestine and colon after 6 h (Figure S4C,D, Supporting Information). Interestingly, *S. paucimobilis* was finally found to be localized inside eWAT after 6 h to 24 h of administration (Figure S4E, Supporting Information). Together, these results revealed that *S. paucimobilis* is translocated from the gut into the circulation and colonizes in eWAT after LPS treatment.

### 2.4 LPS Decreases the level of HETEs, the Main Metabolites of Arachidonic Acid Metabolism after Cold Exposure

**Figure 4:**
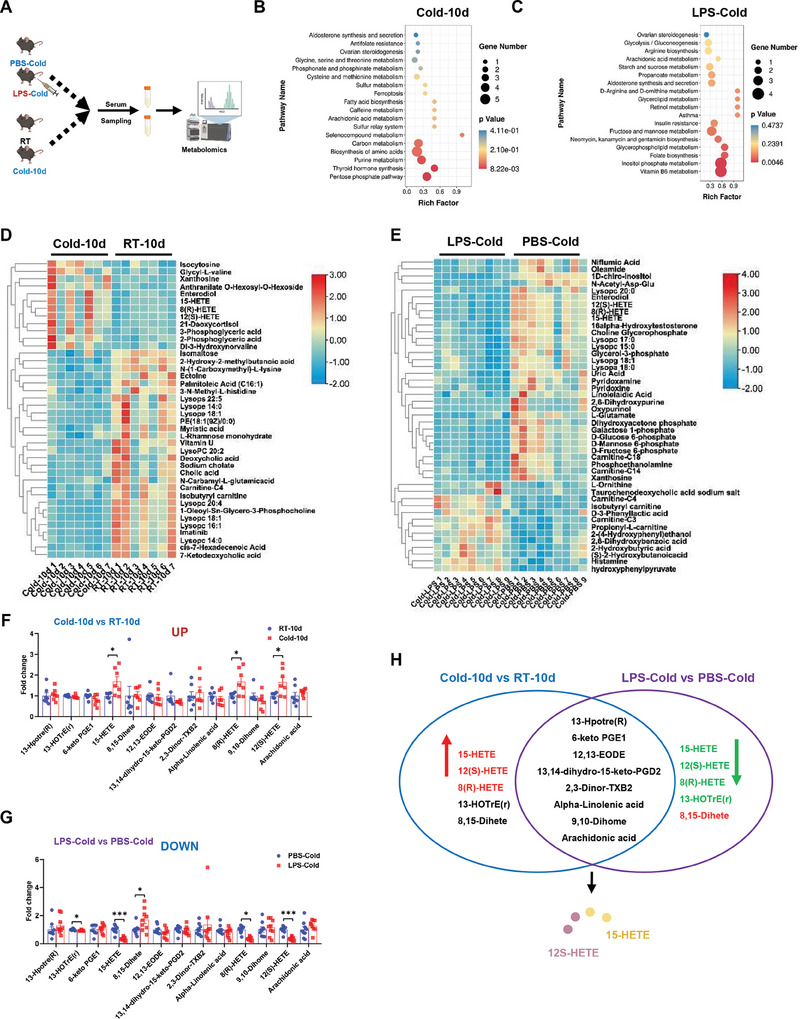
Cold exposure mainly affects the metabolism of arachidonic acid. A) Schematic illustration of metabolomics analysis for serum samples from PBS‐Cold and LPS‐Cold groups. B,C) KEGG pathway enrichment analysis in chronic cold (B) and LPS‐Cold (C) groups. D,E) Heatmap clustering of different metabolites in chronic cold (D) and LPS‐Cold (E) groups. F,G) Analysis of metabolites of ARA in RT, chronic cold, PBS‐Cold, and LPS‐cold treatment, *n* = 7; *n* = 9. H) Schematic illustration of election strategy in key metabolites. *p < 0.05 and **p < 0.01.

To clarify the mechanism underlying the impairing effect of LPS on adaptive thermogenesis, a metabolomic analysis was employed to identify the key metabolites in response to LPS and normal cold treatment (**Figure** **4**A). First, we identified a total of 45 metabolites under LPS treatment and 40 metabolites under normal chronic cold treatment with significant changes for further analysis (Figure 4B,C). Then, we focused on enriched pathways involved in lipid metabolism. Arachidonic acid (ARA) metabolism is one of the important biological processes in thermogenesis,^[^ ^22^ ^]^ which was significantly altered by cold and LPS treatment (Figure 4D,E). Furthermore, the metabolites in ARA metabolism that increased under normal cold treatment but decreased under cold exposure with LPS treatment were identified as potentially core metabolites. Two downstream metabolites of ARA, 15‐HETE and 12S‐HETE, were notably upregulated under cold treatment and significantly downregulated under LPS treatment (Figure 4F–H). These observations indicated that 15‐HETE and 12S‐HETE are potential targets for LPS‐induced defective thermogenesis.

### 2.5 *S. paucimobilis* Impairs Cold Adaptation by Inhibiting 15‐HETE Production and Energy Metabolism

**Figure 5:**
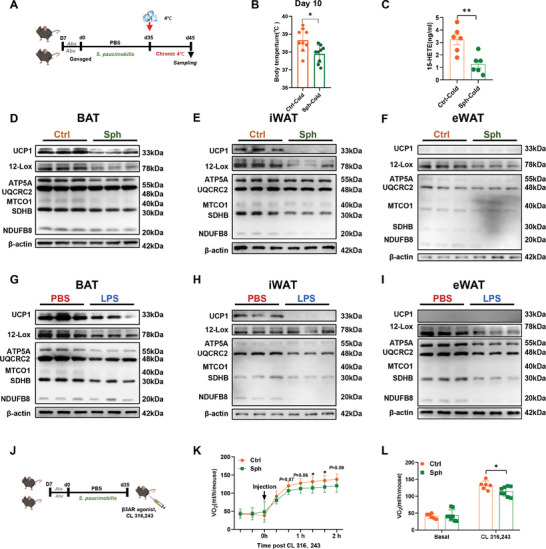
*S. paucimobilis* inhibits adaptive thermogenesis. A) Schematic illustration of *S. paucimobilis* gavage experiment following chronic cold treatment. B) Alterations in body temperature of Ctrl‐Cold and Sph‐Cold groups on day 10 of cold stimulation, *n* = 9. C) Alterations of serum 15‐HETE level in PBS‐gavaged mice with cold treatment (Ctrl‐Cold) group and *S. paucimobilis*‐gavaged mice with cold treatment (Sph‐Cold) groups, *n* = 6. D–F) Western blot analysis of UCP1, 12‐LOX, and total OXPHOS in BAT (D), iWAT (E), and eWAT (F) of Ctrl‐Cold and Sph‐Cold groups. G–I) Western blot analysis of UCP1, 12‐LOX, and total OXPHOS in BAT (G), iWAT (H), and eWAT (I) of PBS‐Cold and LPS‐Cold groups. J) Schematic illustration of *S. paucimobilis* gavage experiment followed CL 316243 treatment. K,L) Oxygen consumption rate (VO_2_) after CL316243 administration at room temperature, *n* = 6–8. * *p* < 0.05 and ** *p* < 0.01.

To investigate the role of *S. paucimobilis* in response to HETEs metabolism and thermogenesis, mice were gavaged daily with freshly prepared live *S. paucimobilis* (1 × 10^9^ live bacteria per day per mouse) for five weeks after depletion of intestinal microbes using broad‐range antibiotics (Abx) administered through drinking water for one week (**Figure** **5**A). There was no change in food intake and body weight (Figure S5A,B, Supporting Information), and a significant decrease was observed in core body temperature in *S. paucimobilis*‐gavaged mice on 10 days but no alterations in 48 h (Figure 5B; Figure S5C, Supporting Information). Next, *S. paucimobilis* strongly inhibited the release of 15‐HETE (Figure 5C) in serum but led to no change in 12S‐HETE (Figure S5D, Supporting Information) after cold exposure for 10 days followed by gavage of *S. paucimobilis*, indicating that *S. paucimobilis* is related to the production of 15‐HETE in vivo. These results indicated that *S. paucimobilis* may act as a mediator in chronic cold treatment.

To clarify the effect of *S. paucimobilis* on adaptive thermogenesis, we further assessed the level of key thermogenesis‐related proteins in adipose tissues. Mitochondrial matrix is the key locale for coordinating the activity of the OXPHOS complexes and UCP1 protein‐mediated uncoupling, which together maintain the thermogenic program of brown adipocytes. Accordingly, the expression of UCP1 and level of OXPHOS complex proteins in mitochondria in the BAT (Figure 5D,G; Figure S5G,J, Supporting Information) and iWAT (Figure 5E,H; Figure S5H,K, Supporting Information) decreased after cold treatment in *S. paucimobilis*‐gavaged mice and LPS‐treated mice. Weak expression of UCP1 and decreased level of OXPHOS complexes was observed in the eWAT in *S. paucimobilis*‐gavaged mice and LPS‐treated mice (Figure 5F,I; Figure S5I,L, Supporting Information). These findings indicate that LPS and *S. paucimobilis* impair the adaptive thermogenesis without alterations in bodyweight and food intake (Figure S5A,B,E,F, Supporting Information). Interestingly, 12‐LOX, the key enzyme that catalyzes ARA to 15‐HETE, was dramatically decreased in all adipose tissues (Figure 5D–I), which is consistent with the level of 15‐HETE in serum, suggesting that *S. paucimobilis* inhibits 15‐HETE production by reducing 12‐LOX.

Next, *S. paucimobilis*‐gavaged mice were intraperitoneally injected with the β3 adrenergic receptor agonist CL316243 (CL) to mimic cold‐activated energy expenditure at thermoneutrality (Figure 5J). Consistent with previous findings, the *S. paucimobilis*‐gavaged mice displayed a significant reduction of whole‐body oxygen consumption in response to CL stimulation (Figure 5K,L). These results suggested that *S. paucimobilis* inhibits adaptive thermogenesis by suppressing 15‐HETE production.

### 2.6 *S. paucimobilis* Reduces the Secretion of 15‐HETE from eWAT

**Figure 6:**
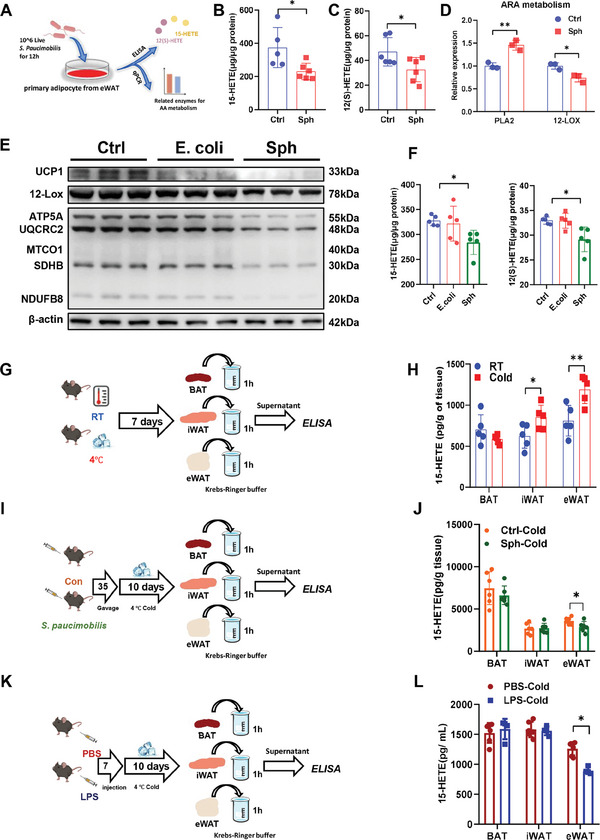
*S. paucimobilis* inhibits the production and release of 15‐HETE. A–C) Co‐culture of live *S. paucimobilis* and primary adipocytes from eWAT (A). 15‐HETE (B) and 12(S)‐HETE (C) level in supernatants of Ctrl and Sph groups, *n* = 6. D) Relative expression of genes related to ARA metabolism, *n* = 3. E) Western blot analysis of UCP1, 12‐LOX, and OXPHOS in differentiated C3H10t1/2 cells treated by PBS, ETEC, or Sph. F) Alterations of supernatants 15‐HETE and 12(S)‐HETE levels in differentiated C3H10t1/2 cells treated with PBS, ETEC, or Sph, *n* = 5. G,I,K) Schematic diagrams of experiments of the source of 15‐HETE in vivo in cold exposure (G), Sph treatment (I), and LPS treatment (K). H,J,L) Alterations of serum 15‐HETE level in various adipose tissues. * *p* < 0.05 and ** *p* < 0.01.

To explore the role of *S. paucimobilis* in regulating 15‐HETE production from adipose tissue, we performed an in vitro co‐culture of live *S. paucimobilis* and adipocytes separated from eWAT (**Figure** **6**A). Interestingly, treatment with live *S. paucimobilis* decreased the cellular content of 15‐HETE and 12S‐HETE (Figure 6B,C). Consistently, the mRNA expression level of *12‐LOX* was dramatically decreased by *S. paucimobilis* treatment (Figure 6D). The level of  *Phospholipase A2 (Pla2)*, an enzyme that catalyzes cell membrane phospholipids to ARA, showed a compensatory increase under *S. paucimobilis* treatment (Figure 6D). To further detect the effect of *S. paucimobilis* on thermogenesis in vitro, differentiated C3H10t1/2 cells were co‐cultured with *S. paucimobilis* or *E. coli*. It could be observed that *S. paucimobilis* significantly inhibited the expression of UCP1, 12‐LOX and OXPHOS complex proteins (Figure 6E; Figure S6A,B, Supporting Information). Accordingly, *S. paucimobilis* remarkably reduced the level of 15‐HETE (Figure 6F). However, the *E. coli* group showed slight changes in all proteins related to thermogenesis as well as the level of 15‐HETE (Figure 6E,F). To further assess whether 15‐HETE is directly produced by adipose tissues and inhibited by LPS or *S. paucimobilis*, we dissected BAT, iWAT, and eWAT under different treatments and incubated these adipose tissue explants in Krebs–Ringer buffer for 1 h, followed by determination of the concentration of 15‐HETE in the supernatant of the buffer (Figure 6G,I,K). Interestingly, there was a significant increase in 15‐HETE released from iWAT and eWAT under cold exposure (Figure 6H), implying that eWAT might also play an important role in response to cold exposure. In addition, significant decreases in the level of 15‐HETE were observed in the eWAT of LPS‐treated and *S. paucimobilis*‐gavaged mice models but not in iWAT or BAT (Figure 6J,L), suggesting that the 15‐HETE produced by eWAT may play a vital role in adaptive thermogenesis. Taken together, these results highlighted an interesting mechanism by which *S. paucimobilis* has direct crosstalk with adipocytes and inhibits the production and release of 15‐HETE, leading to impairment of thermogenesis.

### 2.7 15‐HETE Activates Adaptive Thermogenesis in In Vitro and In Vivo Models

**Figure 7:**
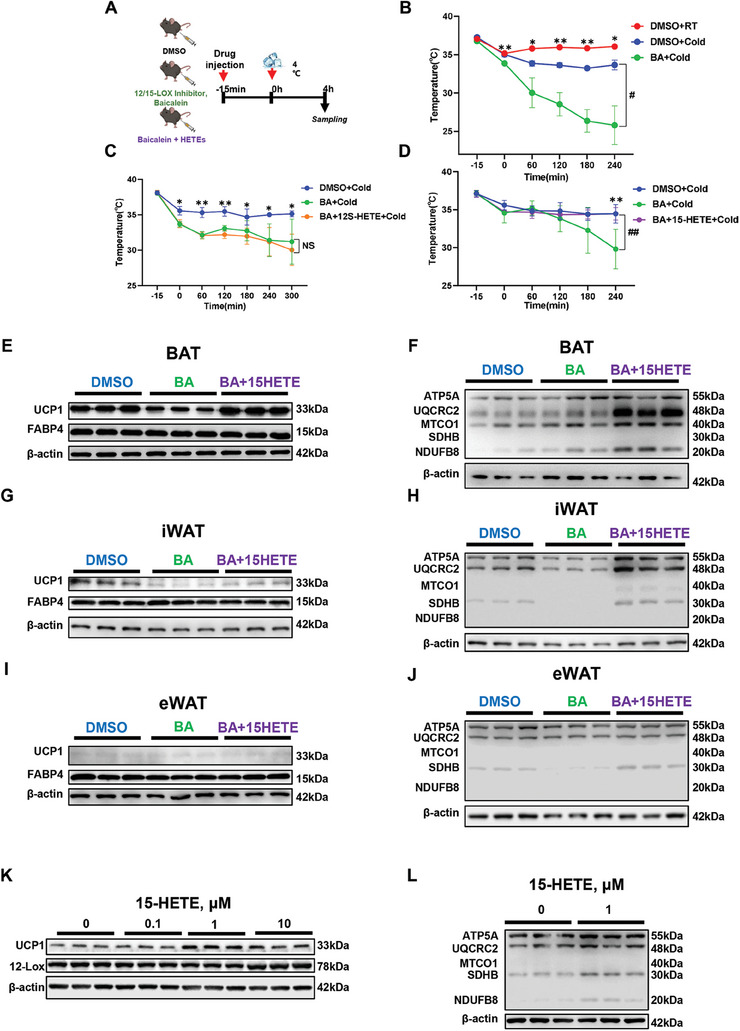
15‐HETE rescues baicalein‐induced ablation of adaptive thermogenesis. A) Schematic diagrams of experiment design. B–D) Change of core body temperature after drug injection in each group, *n* = 3 (B‐C), *n* = 6 (D). * represents the significant difference between DMSO+RT and BA+Cold group, DMSO+Cold and BA+12S‐HETE+Cold group, DMSO+Cold and BA+Cold group; # represents the significant difference between DMSO+Cold and BA+Cold group, BA+Cold and BA+15‐HETE+Cold; * *p* < 0.05 and ** *p* < 0.01, # *p* < 0.05 and ## *p* < 0.01. E–J) Western blot analysis of UCP1, FABP4, in BAT (E), iWAT (G), and eWAT (I) and OXPHOS, in BAT (F), iWAT (H), and eWAT (J) of DMSO, baicalein, and baicalein + 15‐HETE groups. K,L) Western blot analysis of UCP1, 12‐LOX, and total OXPHOS in differentiated C3H10T1/2 cells incubated with 15‐HETE. * *p* < 0.05 and ** *p* < 0.01.

To validate whether 15‐HETE is required for cold adaptation, we co‐injected mice with 15‐HETE or 12‐S‐HETE with baicalein (a 12‐LOX inhibitor, BA)^[^ ^22^ ^]^ for 15 min and then exposed them to 4 °C for 4 h. As shown in **Figure** **7**A–D,  BA strongly inhibited thermogenesis in cold tolerance, which could be rescued by co‐injection with 15‐HETE but not 12S‐ HETE, indicating the critical role of 15‐HETE in adaptive thermogenesis. Consistently, pronounced increases in thermogenic proteins, UCP1 and mitochondrial matrix proteins, were found in the BAT after 15‐HETE injection (Figure 7E,F; Figure S6C, Supporting Information) while only mild changes of UCP1 were found in iWAT (Figure 7G,H; Figure S6D, Supporting Information) and eWAT (Figure 7I,J; Figure S6E, Supporting Information). These findings suggest that 15‐HETE may directly and mainly promote the activity of BAT under acute cold exposure. Moreover, it was found that 15‐HETE could remarkably increase UCP1 and 12‐LOX in a concentration‐dependent manner (Figure 7K; Figure S6F, Supporting Information). Treatment with 15‐HETE also strengthened mitochondrial function in differentiated adipocytes (Figure 7L; Figure S6G, Supporting Information). In summary, these results indicated that 15‐HETE is a vital metabolite required for thermogenesis.

### 2.8 *S. paucimobilis* Inhibits 15‐HETE‐Mediated Phosphorylation of AMPK to Impair Thermogenesis

**Figure 8:**
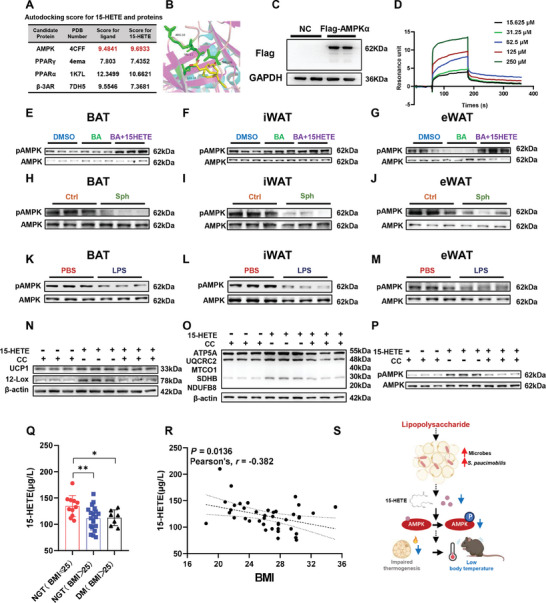
15‐HETE activates AMPK signaling to promote adaptive thermogenesis. A) List of autodocking analysis for 15‐HETE and proteins. B) Molecular docking results of 15‐HETE and AMPK. C) Western blot analysis of overexpressed and purified mouse AMPKα protein. D) SPR assay of different concentrations of 15‐HETE binding responses to AMPKα. E–G) Western blot analysis of pAMPK and AMPK in BAT (E), iWAT (F), and eWAT (G) of DMSO, baicalein, and baicalein + 15‐HETE groups. H–J) Western blot analysis of pAMPK and AMPK in BAT (H), iWAT (I), and eWAT (J) of Ctrl‐Cold and Sph‐Cold groups. K–M) Western blot analysis of pAMPK and AMPK in BAT (K), iWAT (L), and eWAT (M) of PBS‐Cold and LPS‐Cold groups. N–P) Western blot analysis of UCP1 (N), 12‐LOX (N), OXPHOS (O), pAMPK, and AMPK (P) in differentiated C3H10T1/2 cells treated with 15‐HETE or CC. Q) Alterations of plasma 15‐HETE level in individuals with different BMI, *n* = 7‐20. NGT: Normal Glucose Tolerance, DM. R) Scatter‐plots showing a negative correlation between BMI and the level of 15‐HETE. Data are presented as mean ± SD. * *p* < 0.05 and ** *p* < 0.01. S) Schematic diagram illustrating *S. paucimobilis*‐impaired adaptive thermogenesis in response to LPS.

To identify the potential targets of 15‐HETE, we performed autodocking to validate the predicted binding model between thermogenesis‐related proteins and 15‐HETE. Notably, there was a good shape match with high score between AMPK and 15‐HETE (**Figure** **8**A,B). We next examined the binding of AMPK to 15‐HETE. Surface plasmon resonance (SPR) measurement further generated an estimated dissociation constant (KD) value of 100 µM (Figure 8C,D; Figure S7A,B, Supporting Information), suggesting a potential direct interaction between 15‐HETE and AMPK. In terms of AMPK signaling, 15‐HETE treatment for 4 h significantly rescued the decrease of pAMPK in BAT (Figure 8E) and eWAT (Figure 8G) in the BA treatment group, but only resulted in slight changes of that in iWAT (Figure 8F; Figure S7C, Supporting Information). Accordingly, administration of *S. paucimobilis* inhibited the level of pAMPK in BAT and iWAT during long‐term cold exposure (Figure 8H–M; Figure S7D,E, Supporting Information). In differentiated adipocytes, the expression of UCP1 and 12‐LOX was suppressed by compound C (CC), an inhibitor of AMPK, but increased by 15‐HETE (Figure 8N–P; Figure S7F–H, Supporting Information). In general, these results demonstrated that the mediation of 15‐HETE on adaptive thermogenesis may depend on binding and activating of AMPK signaling, which was inhibited by *S. paucimobilis*.

### 2.9 15‐HETE Acts as a Mediator and Biomarker for Overweight and Obesity

Obesity is identified as a chronic inflammatory disease with metabolic syndrome. Because the level of 15‐HETE was increased by cold but decreased by the administration of LPS and *S. paucimobilis*, and 15‐HETE was shown to play a strong thermogenic role, we suspect that 15‐HETE is highly related to obesity. Therefore, we treated diet‐induced obesity (DIO) mice every two days with intraperitoneal injection of 15‐HETE (200 µg kg^−1^) or PBS for two weeks. Although no difference in body weight and food intake or effects on  Glucose tolerance test (GTT) and Insulin tolerance test (ITT) were observed (Figure S8A,C–E Supporting Information), 15‐HETE‐treated DIO mice showed an increased trend of body temperature in the 15‐HETE group (Figure S8B, Supporting Information), suggesting a potential anti‐obesity role of 15‐HETE. Furthermore, we determined the relationship between the plasma level of 15‐HETE and BMI in human subjects. Intriguingly, a remarkably lower level of 15‐HETE was found in overweight (BMI > 25 kg m⁻^2^) and diabetes mellitus (DM) subjects compared with lean individuals (BMI < 25 kg m⁻^2^) (Figure 8Q). Importantly, we identified a significant negative correlation between 15‐HETE and BMI (Figure 8R). These results indicated that a decrease in 15‐HETE may be a risk factor and regulator for chronic inflammation such as overweight and obesity.

## 3 Discussion

The mechanism for chronic inflammation‐mediated defective BAT activation and iWAT browning remains poorly understood. This study aims to clarify the mechanism underlying the defective adaptive thermogenesis in chronic inflammation. We demonstrate that cold exposure with LPS treatment increases the translocation of *S. paucimobilis* from the gut to eWAT and compromises adaptive thermogenesis as indicated by UCP1 expression level and mitochondrial function. Moreover, we found that *S. paucimobilis* inhibits 15‐HETE production and release in an AMPK signaling‐dependent manner. Collectively, our findings suggest a novel mechanism by which adipose tissue‐resident *S. paucimobilis* inhibits adaptive thermogenesis during cold exposure with LPS treatment (Figure 8S).

Obesity‐related metabolic diseases are associated with a low‐grade inflammatory status triggered by LPS accumulation.^[^ ^9^, ^10^ ^]^ LPS also acts as a key risk factor to inhibit adaptive thermogenesis in obesity.^[^ ^8^, ^15^ ^]^ To demonstrate the mechanism for LPS to compromise thermogenesis, we established a model by LPS injection followed by cold exposure. In line with previous studies,^[^ ^8^, ^14^ ^]^ LPS treatment inhibited the browning of iWAT and reduced the body temperature of the mice, indicating that this model is proper for further studying the underlying mechanism. Many studies have demonstrated the presence of bacteria in adipose tissue under obesity and T2D,^[^ ^13^, ^16^, ^17^ ^]^ which may help explain the high level of LPS in obese subjects^[^ ^10^ ^]^ and dissect the potential mechanism for LPS‐reduced thermogenesis. Here, we report an increase in *Sphingomonas*, an adipose tissue‐resident bacterium, under LPS treatment. *Sphingomonas* has also been detected in adipose tissues of subjects with obesity and creeping fat in previous studies,^[^ ^17^, ^20^, ^23^, ^24^ ^]^ indicating that *Sphingomonas* is a candidate bacterium in response to LPS treatment. Furthermore, in situ localization of *α‐proteobacteria* by CARD‐FISH and tracking of the distribution of *S. paucimobilis* revealed that LPS enhanced the translocation of active bacteria from the gut into eWAT, a visceral fat close to the digestive system. These results indicated that adipose tissue‐resident bacteria might be a target in response to LPS treatment. To determine the causative role of *S. paucimobilis* in LPS‐impaired thermogenesis, we gavaged mice with *S. paucimobilis*, and the mice showed a dramatic decrease in UCP1 and OXPHOS complexes with mitochondrial dysfunction. In line with the results reported above, *S. paucimobilis*‐gavaged mice had lower energy expenditure compared with the control mice, indicating the metabolism regulatory effect of *S. paucimobilis*. Thus, it can be speculated that LPS may induce defective thermogenesis by *S. paucimobilis*.

We identified a significant decrease in two novel metabolites, 15‐HETE and 12S‐HETE, involved in ARA metabolism from serum metabolites with or without LPS treatment through lipidomics analysis. Accordingly, a significant decrease in 12‐LOX (an enzyme for producing 15‐HETE and 12S‐HETE) was also observed in all the adipose tissues from in vitro and in vivo models. Interestingly, 12‐LOX catalyzed metabolites improve cold adaptation and glucose metabolism.^[^ ^22^, ^25^ ^]^ Although 12S‐HETE is a widely studied and characterized lipid,^[^ ^22^, ^26^ ^]^ and previous studies have reported a significant increase in 12S‐HETE under acute and chronic cold exposure,^[^ ^22^ ^]^ *S. paucimobilis* gavage did not significantly alter the 12S‐HETE level in mice. In addition, intraperitoneal injection of 12S‐HETE did not rescue the abolishment of 12‐LOX inhibitor‐induced defective adaptive thermogenesis, which is in accordance with previous report of slight changes in 12S‐HETE in response to 12‐LOX block treatment.^[^ ^22^ ^]^ In this regard, 12S‐HETE may play an important role in glucose uptake and insulin resistance but not in adaptive thermogenesis.^[^ ^22^, ^27^ ^]^ Another study showed an unchanged level of 15‐HETE in mice and humans acutely treated with Mirabegron.^[^ ^22^ ^]^ However, we found that 15‐HETE was significantly increased under chronic cold exposure. Particularly, WAT may represent a potential source of oxylipins as previously reported.^[^ ^25^ ^]^ The volume of visceral fat is always associated with metabolic inflammation.^[^ ^28^, ^29^ ^]^ Intriguingly, our results demonstrated that eWAT and iWAT but not BAT are the main tissues producing 15‐HETE, and the 15‐HETE level decreased in eWAT of LPS‐treated and *S. paucimobili*s‐gavaged mice models, indicating an indispensable role of visceral fat in activating thermogenesis. Moreover, injection of 15‐HETE rescued 12‐LOX blocking‐induced defective thermogenesis and presented a strong effect to defend body temperature loss. In accordance with these findings, significant increases in UCP1 were observed in BAT in response to 15‐HETE treatment, which may establish a crosstalk between eWAT and BAT via the release of lipid mediators.

We further conducted autodocking and SPR of 15‐HETE to confirm the molecular targets of 15‐HETE. The results indicated that AMPK signaling may be its direct target. Since AMPK signaling in thermogenesis has been well clarified,^[^ ^30^ ^]^ we speculate that 15‐HETE may induce UCP1 expression via AMPK signaling. Consistently, in LPS‐treated or *S. paucimobilis*‐gavaged mice models, the phosphorylation of AMPK was significantly decreased in adipose tissues. These results provide mechanistic insights into endogenous lipid mediator‐induced adaptive thermogenesis in an AMPK‐dependent manner. Furthermore, the lower level of 15‐HETE in obese and overweight subjects and the effect of 15‐HETE to elevate body temperature in DIO mice indicate the possibility of activating thermogenesis to ameliorate obesity.

## 4 Conclusion

In summary, our results demonstrated an important role of adipose tissue‐resident *S. paucimobilis* in LPS‐compromised adaptive thermogenesis (Figure 8S). We clarified that 15‐HETE is a metabolite produced by eWAT, which is inhibited in LPS treatment and *S. paucimobilis* gavage, and elevates BAT activity by activating AMPK signaling. Collectively, our findings indicate an underlying mechanism for LPS‐compromised adaptive thermogenesis and novel therapeutic approaches for promoting thermogenesis to treat obesity.

## 5 Experimental Section

### Ethics Statement

All animal procedures were approved by the Institutional Animal Care and Use Committee of Huazhong Agricultural University (HZAUMO‐2020‐0106). All samples from human individuals were approved by Beijing Chao‐yang Hospital, Capital Medical University (No. 2022‐ke‐266).

### Study with Overweight/Obese and Lean Human Subjects

A cohort of 38 individuals was selected from Beijing Chao‐yang Hospital (7 females and 31 males) to represent a wide range of BMI (18.42–35.16 kg m⁻^2^), who were categorized as lean (BMI < 25 kg m⁻^2^; *n* = 11; 7 male (M), 4 female (F)), overweight/obesity (BMI > 25 kg m⁻^2^; *n* = 20; 17 M, 3 F) or DM (BMI > 25 kg m⁻^2^; *n* = 7; 7 M, 0 F), and glucose metabolism parameters (Fasting plasma glucose 3.12–10.16 mmol L⁻^1^; HbA1c: 4.7%–10.1%). In the subgroup of lean and overweight/obesity subjects, all individuals had normal glucose tolerance (NGT), and in the DM group, all 7 individuals had T2D. Collection of human biomaterials, plasma analyses, and phenotyping were approved by the ethics committee of the Beijing Chao‐yang Hospital, Capital Medical University (No. 2022‐ke‐266), and all individuals gave written informed consent before taking part in the study.

### Mice and Treatment

All specific pathogen‐free (SPF) and germ‐free male C57BL/6J mice were fed in 12/12 h day/night cycles in the animal center of Huazhong Agricultural University (Wuhan, China), and provided with a standard laboratory diet and clean water. Each group of mice was kept in separate cages. All mice were allocated to experimental groups based on their body weight to ensure equal starting points. All mice were kept one per cage at 4 °C for cold exposure treatment,^[^ ^31^ ^]^ and the bacterial transplantation experiment was conducted on 8‐week‐old mice which were orally gavaged with *S. paucimobilis* (10^9^ colony forming units (CFU)/day).

For the LPS‐treatment, 8‐week‐old C57BL/6J were intraperitoneally injected every other day for two weeks prior to 4 °C cold treatment. Mice were injected intraperitoneally every other day with 300 µg kg^−1^ (based on body weight of mice) LPS diluted in PBS solution or with vehicle (PBS, 4 mL kg⁻^1^, based on body weight of mice). LPS from *E. coli* 055: B5 were purchased from Sigma (Sigma, L2880). E.g., a mouse with 25 g body weight was injected with 7.5 µg LPS. Body weight and food intake were monitored. Serum was collected. All mice were allowed ad libitum access to water and food. After two weeks for injection, cold treatment was performed for all mice. The core body temperature was measured by rectal thermometer after the cold treatment.^[^ ^8^, ^14^ ^]^

For detecting the level of 15‐HETE in different adipose tissues, by using an ex vivo tissue incubation method, BAT, iWAT, and eWAT were dissected from 8‐week‐old C57BL/6J male mice individually housed at 4 °C or room temperature for 7 days and incubated at 37 °C in 300 µL of Krebs–Ringer solution (pH 7.4) (Solarbio, G0430) for 1 h. Same ex vivo experiments were employed for detecting the level of 15‐HETE in adipose tissues from LPS‐induced and *S. paucimobilis*‐gavaged mice. Then, the tissues were discarded and a Mouse 15‐HETE ELISA Kit (LabRe, CAS: LB30577B) was used to detect the 15‐HETE level in the supernatant of Krebs‐Ringer solution.

For 15‐HETE injection in DIO mice, 17‐week‐old DIO C57BL/6J male mice were injected intraperitoneally with 200 µg kg^−1^ body weight of 15‐HETE diluted in PBS solution or with vehicle (PBS) every other day for seven times. Body weight, food intake, and body temperature were monitored. After the completion of all injections, the GTT was performed in 6 h‐fasted DIO mice. The blood sample was collected from the tail of fully conscious mice, followed by intraperitoneal injection of glucose (2.0 g kg⁻^1^ body weight), and blood glucose concentrations were measured at 0, 15, 30, 45, 60, 90, and 120 min after injection. For the ITT, the blood samples from DIO mice fasted for 6 h (7AM to 1PM) were collected from the tail of fully conscious mice. Insulin (1 U kg⁻^1^ body weight) (Pricella, CAS: PB180432) was administered by intraperitoneal injection, and blood glucose concentrations were determined at 0, 15, 30, 45 and 60 min after injection.

### 16S rRNA Gene Analysis

The tissue microbes were analyzed as previously described.^[^ ^32^ ^]^ Briefly, total bacterial DNA was extracted using a DNA stool mini kit (Tiangen, Beijing, China). The V3–V4 region of bacterial 16S rRNA genes was amplified and sequenced by the Shanghai Personal Biotechnology Limited Company (Shanghai, P. R. China) using an Illumina MiSeq (Illumina, USA) sequencing platform. The sequencing reads were analyzed by the QIIME2 (quantitative insights into microbial ecology, http://www.qiime.org) analysis pipeline as previously described. In brief, FASTA quality files and a mapping file indicating the barcoded sequence corresponding to each sample were used as inputs. The reads were split by samples according to the barcode. Taxonomical classification was performed using the RDP‐classifier, and an operational taxonomic unit (OTU) table was created. Closed reference OTU mapping was employed using the RDP database (http://rdp.cme.msu.edu). Sequences sharing 97% nucleotide sequence identity in the V3–V4 region were binned into operational taxonomic units (97% ID OTUs). α‐Diversity (Shannon index and observed species) and β‐diversity (Jaccard distance) were calculated using QIIME2. Differential species associated with particular interventions were identified by LEfSe with the effect size threshold of 3.5.

### Catalyzed Reporter Deposition Fluorescence In Situ Hybridization (CARD‐FISH)

Visualization of bacterial cells in the tissue was carried out using CARD‐FISH as previously described.^[^ ^17^ ^]^ Briefly, the tissues of mice were fixed in 4% paraformaldehyde (Sangon Biotech, E672002), then embedded in paraffin and sectioned. Deparaffinized sections were sequentially treated with permeability mixture buffer (Proteinase K buffer (Biosharp, BS080), SDS buffer, lysozyme buffer (Biosharp, BS184) and achromopeptidase buffer) for 1 h at 37 °C to achieve permeabilization. The slides were incubated in hybridization buffer (Sodium chloride, Tris‐HCL, Triton X‐100 (Aladdin, T109026), Maleic acid (Biosharp, BS930) and Dextran sulfate (Macklin, D806297)) with HRP‐labeled CARD‐FISH probes (0.17 ng mL⁻^1^) for 3 h at 37 °C in a humidified chamber, and then washed gently three times in wash buffer and 1 × PBS for 15 min at room temperature. CARD‐FISH was performed by incubating the sections for 20 min at 37 °C in amplification buffer (PBS, Dextran sulfate, Formamide (Sigma‐Aldrich, F9037), Maleic acid (Macklin, P823909)). The samples were then washed three times with 1 × PBS and stained with DAPI (1 µg mL⁻^1^) (Beyotime, P0131) for 10 min at room temperature. The mixture probes for total bacteria detection were as follows: EUB‐338I: 5′‐GCTGCCTCCCGTAGGAGT ‐3′; EUB‐338II: 5′‐GCAGCCACCCGTAGGTGT‐3′; EUB‐338III: 5′‐ GCTGCCACCCGTAGGTGT‐3′. For α‐proteobacteria detection, probes were as follows: 5′‐ GAATTTCACCTCTACACT ‐3′.^[^ ^18^ ^]^ Images were acquired on a Leica inverted fluorescence microscope Leica inverted fluorescence microscope (Leica, DMi8, Germany).

### Tissue Clearing and Imaging

To clear the mouse tissues, fixed tissue samples were incubated with 10 mL of FUnGI clearing solution at 4 °C in the dark overnight. The FUnGI clearing agent (50% glycerol, 2.5 M fructose, 2.5 M urea, 10.6 mM Tris Base, 1 mM EDTA) was prepared as previously described.^[^ ^33^ ^]^ After the tissues became transparent, they were mounted on slides and stained with DAPI (1 µg mL⁻^1^) for microscope analysis. Fluorescence 3D images were acquired as previously described.^[^ ^21^, ^33^ ^]^ Briefly, labeled *S. paucimobilis* was directly visualized using a STORM Laser scanning confocal microscope (Nikon, AXR NSPARC, Japan). Samples were excited at 468 nm for DAPI and 556 nm for TAMRA, and emissions were detected using corresponding emission filters. For multiphoton imaging, tile scans of Z‐stacks were acquired at an optical section resolution of 512 × 512 using a 25× 1.0 NA objective. DAPI (blue) staining was used for nucleus and bodipy (green) staining was used for lipid droplets. These tissue sections were stacked up with a total of 60 sections with a total volume of 371 × 371 × 60 µm^3^ and positive dots were collected from the spatial volume of tissue for further statistical analysis.

### Histology

Adipose tissue samples (inguinal and epididymal white fat) were fixed immediately in 4% paraformaldehyde. Paraffin‐embedded adipose tissues were sectioned into 6‐µm slides and stained with hematoxylin–eosin (H&E) using standard techniques.^[^ ^8^ ^]^

### Serum and Plasma Parameters

The whole blood collected from the mice before sacrifice was separated for plasma by centrifugation at 1200 × g for 10 min at 4 °C. LBP, 15‐HETE and 12S‐HETE were measured by enzyme linked immunosorbent assay (ELISA) (Meimian Biotechnology) according to the manufacturers’ instructions.^[^ ^20^ ^]^

### Metabolomics Analysis

The serum samples (100 µL per tube) were placed in tubes and resuspended with prechilled 80% methanol and 0.1% formic acid by well vortex. Then, the serum samples were incubated on ice for 5 min and centrifuged at 15000 × *g* and 4 °C for 20 min. Some of the supernatants were diluted to a final concentration containing 53% methanol by water for LC‐MS. The samples were subsequently transferred to fresh Eppendorf tubes and then centrifuged at 15000 × g and 4 °C for 20 min. Finally, the supernatants were injected into the LC‐MS/MS system for analysis.^[^ ^34^, ^35^ ^]^ LC‐MS/MS analyses were performed using an ExionLC AD system (SCIEX) coupled with a QTRAP 6500+ mass spectrometer (SCIEX) at Novogene Co., Ltd. (Beijing, China). The detection of the experimental samples using MRM (multiple reaction monitoring) was based on the Novogene in‐house database.

### Analysis of Non‐Shivering Thermogenesis

To test the non‐shivering thermogenic (NST) capacity of *S. paucimobilis*‐treated mice raised at room temperature, we determined O_2_ consumption and CO_2_ production in a thermally and humidity‐controlled environment using a multi‐channel small animal metabolism monitoring system (Oxymax/ CLAMS, Columbus instruments, Columbus, OH) for 12 h a day and 12 h a night. Mice were housed in single cages with ad libitum access to food and water. To test the NST ability of *S. paucimobilis*‐treated mice, the β3 receptor agonist CL316243(CL) (Sigma, USA) was administered to simulate cold exposure. Metabolic rates upon administration of CL were studied using a multi‐channel small animal metabolism monitoring system. Mice were anesthetized with pentobarbital (75 mg per kg body weight, intraperitoneal injection), which does not inhibit NST. Basal metabolic rates were monitored for the subsequent 40 min until reaching steady state. Then, CL was injected intraperitoneally using a dosage of 1 mg per kg of body weight. The subsequent increase in metabolic rates was monitored for 2 h. Data were presented as VO_2_ (mL min^−1^).^[^ ^36^ ^]^

### Primary Cell Culture and Differentiation

For stromal vascular fraction (SVF) isolation, dissected adipose tissues were minced with scissors and digested for 45 min at 37 °C in digestion buffer (25 mM NaHCO_3_, 12 mM KH_2_PO_4_, 1.2 mM MgSO_4_, 4.8 mM KCl, 120 mM NaCl, 1.4 mM CaCl_2_, 5 mM glucose, 2.5% BSA, 1% penicillin–streptomycin, 1 mg mL⁻^1^ collagenase type 1(C6885‐1G, Sigma‐Aldrich), pH = 7.4). An equal volume of culture medium (high‐glucose DMEM medium (61965026, Gibco) supplemented with 20% fetal bovine serum (FBS) and 1% penicillin–streptomycin) was added and digested cell mixture was filtered through 70 µm cell strainers (Corning, 431752, Corning, NY, USA) to remove undigested tissues and then centrifuged for 8 min at 1500 rpm. The supernatant was removed and the SVF pellet was resuspended in culture medium. Four hours after incubation, cell medium was replaced with fresh 10% FBS culture medium. SVF and mouse mesenchymal stem cell line C3H10t1/2 cells were seeded into a plate and differentiated as previously described.^[^ ^37^ ^]^

For adipogenic differentiation, cells were treated with 1 µM Dexamethasone, 0.5 mM isobutyl‐methylxanthine, 10 µg mL⁻^1^ insulin and 100 µM indometacin. After 2 days, the cells were transferred to 10% FBS medium containing only 10 µg mL⁻^1^ insulin and maintained in this medium for 2 days; subsequently, cells were maintained in 10% FBS for another 2 days.

For inhibitors treatment experiments, differentiated mature adipocytes were pre‐incubated with CC (10 µM) (MedChemExpress, HY‐13418A) for 2 h, followed by 15‐HETE (1 µM) treatment for 24 h.

### Bacterial Culture

*S. paucimobilis* (ATCC 29837) was purchased from the China General Microbiological Culture Collection Center (Beijing, China). *S. paucimobilis* was cultured for 2 days in basal nutrient broth at 30 °C. When the bacterial concentration reached 10^9^ CFU, bacteria were transferred to 4 °C for storage. The *S. paucimobilis* was washed in sterile PBS and administered by oral gavage of 1×10^9^ CFU per mouse. Bacteria were freshly prepared every week.

### Immunofluorescence

Intestinal samples for immunofluorescence were incubated in 4% paraformaldehyde for at least 24 h and embedded in paraffin. Paraffin blocks were sliced into 6‐µm slides, treated with DAKO retrieval solution (pH = 9) for 30 min^[^ ^17^ ^]^ and stained with anti‐mouse LPS (Abcam, ab35654) and LTA antibody (Abclonal,  A1552). The primary antibody was incubated at 4 °C overnight, and slices were then incubated with the appropriate fluorophore‐conjugated secondary antibody.^[^ ^38^, ^39^ ^]^ Before imaging, nuclei were counterstained with DAPI or Hoechst.

### Fluorescent D‐Amino Acid (FDAA) Loading and Tracking

FDAA loading and tracking were performed as previously described.^[^ ^21^ ^]^ Briefly, *S. paucimobilis* was cultured in basal nutrient broth at 30 °C in an aerobic chamber till mid‐exponential phase. TAMRA‐amino‐D‐alanine (TADA 0.75 mM; presented by Dr. Wei Wang from Shanghai Jiao Tong University) was then added to the culture medium for overnight labeling. The bacteria were then washed with dPBS twice, and re‐suspended in dPBS for transplantation. The germ‐free mice were administered with FDAA‐labeled bacteria (10^9^ CFU) by oral gavage. The recipient mice received 100 µL of 1 M NaHCO_3_ by gavage 30 min before bacterial transplantation to neutralize gastric acid. After 6 h, 12 h and 24 h, the ileum, colon, and eWAT were collected and fixed in 4% paraformaldehyde at 4 °C in the dark without shaking.

### Preparation of 15‐HETE and 12S‐HETE

12S‐HETE and 15‐HETE purchased from Aladdin (CAS: 54397‐83‐0; CAS: 71030‐36‐9) were provided in the form of an ethanol solution. In order to change the solvent, the ethanol was evaporated under a mild nitrogen flow and the resulting pure oil was immediately dissolved in PBS.

### Cold Tolerance Tests

Eight‐week‐old C57BL/6J male mice were intraperitoneally injected with 50 µL of 50 mg k^−1^g 12/15‐LOX inhibitor baicalein (Aladdin, CAS:491‐67‐8) or DMSO, 15 min before placing the mice at 4 °C for 4 h. In another set of experiments, baicalein was co‐injected with 200 µg k^−1^g of 15‐HETE or 12S‐HETE 15 min before starting the cold exposure. Rectal temperature measurements were conducted every hour. After the experiments, serum, BAT, iWAT and eWAT were collected.

### Protein Expression and SPR

FLAG‐tagged AMPKα was expressed in a 10‐cm dish of HEK293T cells (grown to 80% confluence) transfected with 10 µg of indicated plasmids per dish for 48 h. After 48 h, cells were collected by centrifugation at 2000 × g, and then lysed with 1 mL of ice‐cold lysis buffer. The lysates were then sonicated and centrifuged at 13000 × g for another 30 min at 4 °C. Anti‐FLAG magnetic beads (1:100, balanced in lysis buffer) were added into the supernatant and mixed overnight at 4 °C. The beads were then washed with 200 times volume of lysis buffer for 3 times at 4 °C. Proteins were then eluted with 100 µL of 3×FLAG peptide (400 µg mL⁻^1^) for another 2 h at 4 °C. Sodium dodecyl sulfate‐polyacrylamide gel electrophoresis (SDS‐PAGE) was conducted to detect the concentration of eluted protein. Different doses of bovine serum albumin (BSA) were indicated as the concentration references.

For SPR analysis, CM5 Sensor Chip was esterified with crosslinking agents EDC and NHS. The purified protein at the concentration of 50 µg mL⁻^1^ in sodium acetate at pH 4.0 was coupled to the surface of the chip, then the remaining reactive carboxyl on the matrix was blocked using 1 M ethanolamine at pH 8.5. The 15‐HETE was dissolved in PBS to 10 mM, and diluted with HBS‐EP buffer solution to 500, 250, 125, 625, 31.25, 15.625, and 7.813 µM, respectively. The SPR experiment was performed using the Biacore T200 SPR (Cytia, Biacore T200) instrument. The injection sample time and velocity were 120 s and 10 µL min^−1^, and the protein dissociation time was 180s. The data were analyzed by the SPR kinetic evaluation software, BIA evaluation.

### Western Blot

The samples were extracted with protein lysis buffer (Beyotime, China) supplemented with protease inhibitor cocktail. Protein concentration was determined using the BCA Kit (Beyotime, China). Proteins (25‐35 µg) were separated on a 10% polyacrylamide precast SDS‐PAGE gel (Bio‐Rad) followed by blotting on PVDF membranes (Millipore Billerica, MA, USA). The membranes were probed with the following antibodies against: 12‐LOX (Santa Cruz, sc‐365194, 1:100), UCP1 (proteintech, 23673‐1‐AP, 1:2000), OXPHOS cocktail (Abcam, ab110413, 1:1000), FABP4 (Abclonal, A11481, 1:750), AMPK (Cell Signaling Technology, AB 10622186, 1:1000), pAMPK (Cell Signaling Technology, AB 331250, 1:1000), β‐actin (Abclonal, AC038, 1:10000); GAPDH (Proteintech, 60004‐1‐Ig, 1:10000), and Flag (Abclonal, ae063, 1:3000).

### Absolute and Relative Real‐Time qPCR

Gene expression analysis was operated as previous described.^[^ ^37^ ^]^ Briefly, total RNA was extracted using the Trizol reagent (Invitrogen, USA) and transcribed into cDNA using a first‐strand cDNA synthesis kit (TOYOBO, Japan). Quantitation of the mRNA level by QPCR was performed on a real‐time PCR system using iTaq Universal SYBR Green Supermix (Bio‐Rad, Richmond, CA, USA). The cycle thresholds (Ct) value of the target gene was normalized to the Ct of the internal reference *β‐actin* gene using the formula “2^−ΔΔCt^”, which yielded relative gene expression level values. The primers used were as follows: *β‐actin*, Forward 5′‐GGCACCACACCTTCTACAATG‐3′, Reverse 5′‐ GGGGTGTTGAAGGTCTCAAAC‐3′; *12‐LOX*, Forward 5′‐ TCCCTCAACCTAGTGCGTTTG‐3′, Reverse 5′‐GTTGCAGCTCCAGTTTCGC‐3′; *PLA2*, Forward 5′‐GCAGGCAGAGCGATATGATG‐3′,^[^ ^22^ ^]^ Reverse 5′‐CAGCTCCGTCTCGATCTTCT‐3′.^[^ ^40^ ^]^

For bacteria copy number analysis, total DNA was purified from adipose tissues as previous described.^[^ ^18^, ^41^ ^]^ Takara2 Premix Ex Taq (Takara, RR390A) kit was used for qPCR analysis, which was performed on a Bio‐Rad real‐time PCR system (Bio‐Rad). *E. coli* DNA was used to establish a standard curve to calculate bacterial DNA concentration in the sample and negative controls were included for the reactions. Absolute qPCR experiment was carried out, 10 µL reaction mix containing Premix Ex Taq (probe qPCR), 750 nM of forward primer, 500 nM reverse primer and 250 nM probe, and 1 µL sample DNA was loaded on the real‐time system. The reaction was programed as follows: denaturation at 94 °C for 10 min, 40 cycles of 94 °C for 15 s, 60 °C for 60 s. Ct values were normalized according to a bacterial standard curve produced with *E. coli* DNA. The primers used for absolute qPCR analysis were as follows: V9 region of the 16S ribosomal RNA gene were amplified with following primers: Forward 5′‐CGGTGAATACGTTCYCGG‐3′, Reverse 5′‐GGWTACCTTGTTACGACTT‐3′, and Probe 5′‐CTTGTACACACCGCCCGTC‐3′.

### TEM

Adipose tissue samples were fixed, washed, and post‐fixed. After which cells were dehydrated and embedded in EPOK 812 (Oukenn, Tokyo, Japan). Then samples were post‐stained with uranyl acetate and lead citrate at room temperature for 20 min. The stained adipose tissue samples were then analyzed using a TEM (H‐600IV; Hitachi High‐Technologies Corp.; Japan). Images were analyzed using IPP v6.0 (Media Cybernetics).

### Statistical Analysis

Statistical analysis was performed using GraphPad Prism software v 8. Data were assessed for normal distribution and plotted in the figures as the mean ± SD. For each figure, *n* = the number of subjects. For comparison of two groups, Student's t‐test was used. For comparison of more than two groups, ANOVA was performed followed by post hoc Bonferroni's multiple comparison test to determine the significance between groups. For linear regression analysis, significance of the correlation was determined by Pearson's test. Statistical difference was considered as significant when *p* < 0.05.

## Conflict of Interest

The authors declare no conflict of interest.

## Author Contributions

Y.Z., R.Y., Z.D., and B.D. are co‐first authors. T.S., Z.M., Y.Z., R.Y., B.D., and Z.D. designed the research and analyzed all of the results; Y.Z., R.Y., Z.D., and B.D. performed animal studies; T.S., B.D. and Y.Z. performed cellular experiments; R.Y. and Y.Z. performed the CARD‐FISH experiment; C.D., Y.W., K.Z., and G.W. contributed to clinical samples and methodology; J.Z., Z.R., and W.L. reviewed the final manuscript. T.S., Y.Z., Y.X., and Z.M. wrote the paper.

## Supporting information



Supporting Information

Supporting Information

## Data Availability

All data are available in the main text or the supporting information. The accession number for 16S rRNA sequence data reported in this paper is NCBI BioProject: PRJNA753964.
