# Supplementary material for: Adipose Tissue‐Resident Sphingomonas Paucimobilis Suppresses Adaptive Thermogenesis by Reducing 15‐HETE Production and Inhibiting AMPK Pathway
Source: Adv Sci (Weinh). 2024 Oct 30;11(47):2310236. doi: 10.1002/advs.202310236 (PMC11653631; doi:10.1002/advs.202310236)
Supplement: Supplementary file 1 — Supporting Information [file ADVS-11-2310236-s002.docx]

**Supporting information**

**Adipose tissue-resident *Sphingomonas paucimobilis* suppresses adaptive thermogenesis by reducing production of 15-HETE and inhibiting AMPK pathway**

*Yucheng Zhu^1#^, Ruiqi Yang^1#^, Zhangchao Deng^1#^, Bohua Deng^1#^, Kun Zhao^2^, Chen Dai^3^, Gang Wei^4^, YanJiang Wang^5^, Jinshui Zheng^6^, Zhuqing Ren^1^, Wentao Lv^7^, Yingping Xiao^7*^, Zhinan Mei^8*^, Tongxing Song^1*^*

**Supplemental Figures**

**
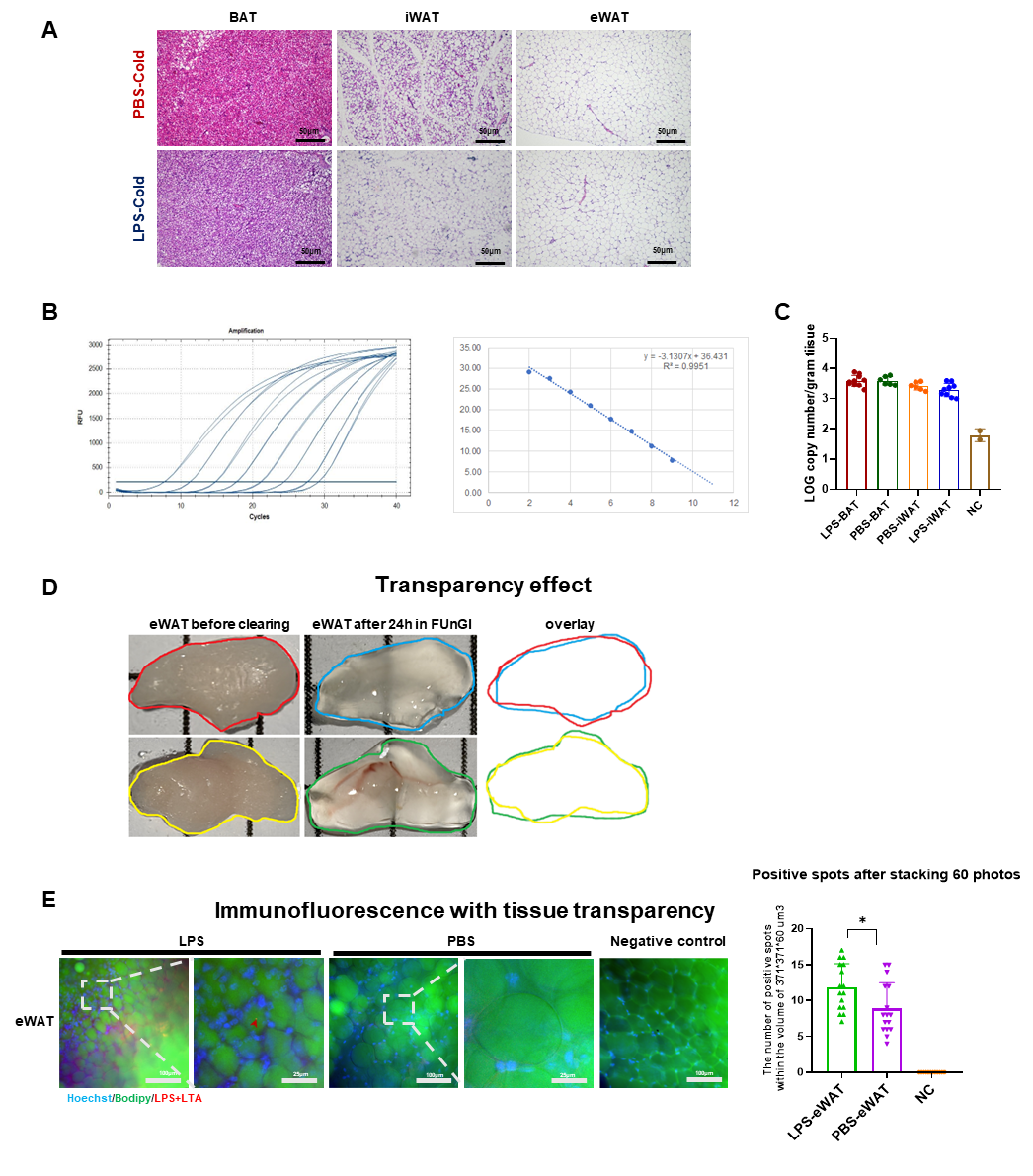
**

**Figure S1. Microbes in adipose tissue.**

(A) Histology of adipose tissues. Scale bar is 50μm.

(B) Standard curves for qPCR.

(C) qPCR for bacteria quantification in iWAT and BAT, n = 6-10. Negative control, NC.

(D)Images of adipose tissue before clearing and 24 h after clearing. The outlines of the adipose tissue (before incubation and 24 h later, respectively) are overlaid. The overlays demonstrate no distortion after clearing with FUnGI.

(E) IF staining of LPS+LTA (red) to show the localization of gram-positive and gram-negative bacteria in eWAT. Hoechst (blue) staining was used for nucleus and bodipy (green) staining were used for lipid droplets. These tissue sections stacked up a total of 60 sections with a total volume of 371*371*60 μm³ and positive dots were collected from the spatial volume of tissue for further statistical analysis, n = 16. Scale bar is 100 μm. Scale bar is 25 μm for enlarged image. * *P* < 0.05; ** *P* < 0.01.

**
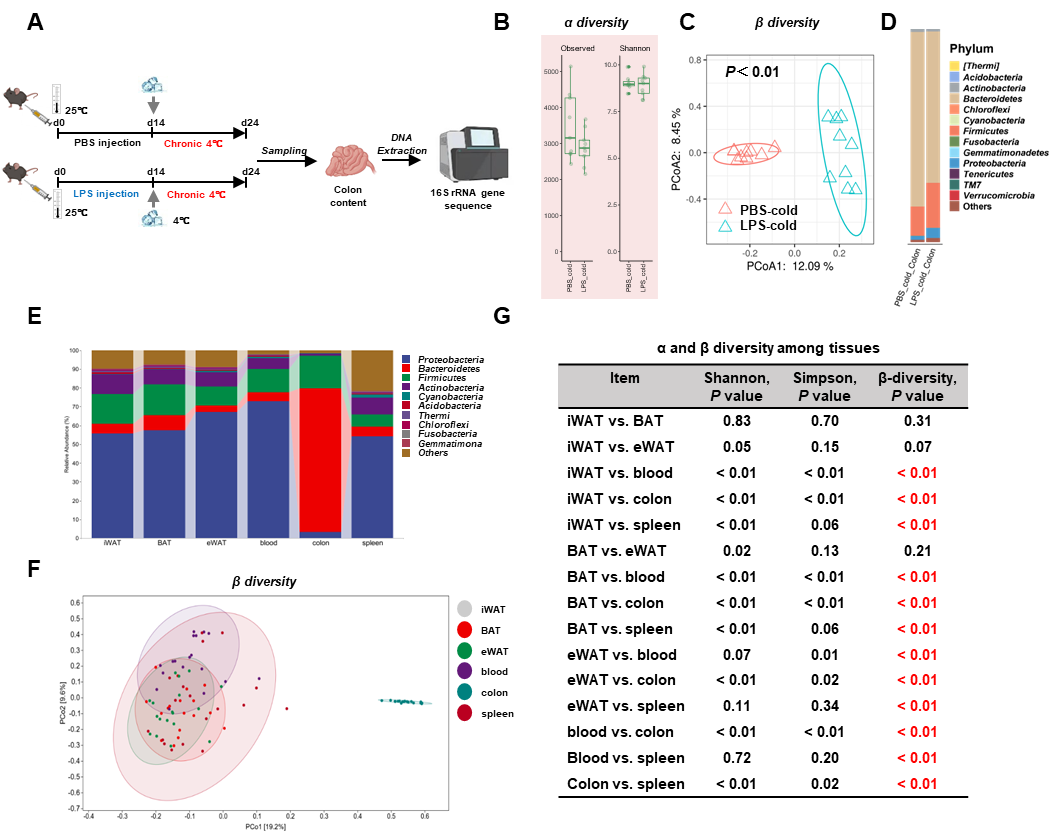
**

**Figure S2. Microbial diversity between adipose tissue and other tissues.**

(A) Schematic diagram of LPS treatment followed by cold exposure experiment.

(B) Observed diversity and Shannon index in colon content at the phylum level. Differences in α-diversity of each group were tested by Wilcoxon rank-sum test. * *P* < 0.05; ** *P* < 0.01.

(C) PCoA based on Jaccard distance revealed microbiota clustering in colon after LPS + chronic cold treatment. The percentage of variation explained by the plotted principal coordinates is indicated in the axis labels. Differences in β-diversity in each group were tested by Adonis. * *P* < 0.05; ** *P* < 0.01.

(D) Relative bacterial abundance at the phylum level in colon of mice in PBS-Cold and LPS-Cold group.

(E) Relative bacterial abundance at the phylum level in different tissues of mice in PBS-Cold and LPS-Cold group.

(F) PCoA based on Jaccard distance revealed microbiota clustering in different tissues after LPS + chronic cold treatment. The percentage of variation explained by the plotted principal coordinates is indicated in the axis labels. Differences in β-diversity in each group were tested by Adonis. * *P* < 0.05; ** *P* < 0.01.

(G) Comparison of tissue microbes among adipose tissues, spleen, blood and colon. * *P* < 0.05; ** *P* < 0.01.


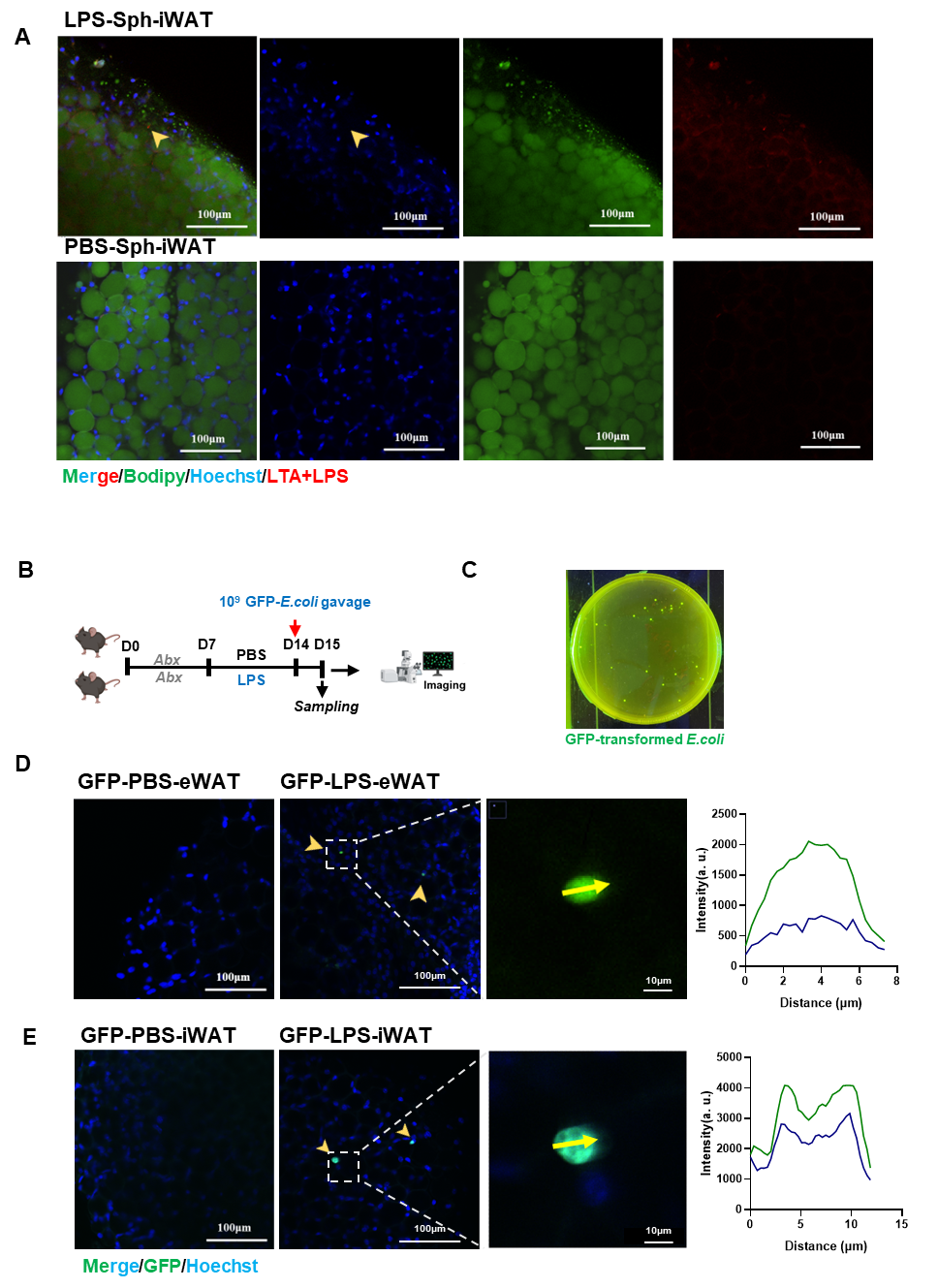


**Figure S3. LPS increases bacterial translocation in eWAT.**

(A) Representative images of iWAT after *S. paucimobilis* (Sph) gavage. The *S. paucimobilis* and nuclei were stained with LPS/LTA antibody (red) and Hoechst (blue) respectively. Scale bar is 100 μm.

(B) Schematic illustration of GFP-transformed *E. coli* gavage experiment.

(C) Image showing GFP-*E. coli* at 488nm wavelength.

(D-E) Representative images of eWAT (D) and iWAT (E) after GFP-*E. coli* gavage. The nuclei were stained with Hoechst (blue). Relative intensity within the indicated yellow area was measured. Scale bar is 100 μm. Scale bar is 10 μm for enlarged image.

**
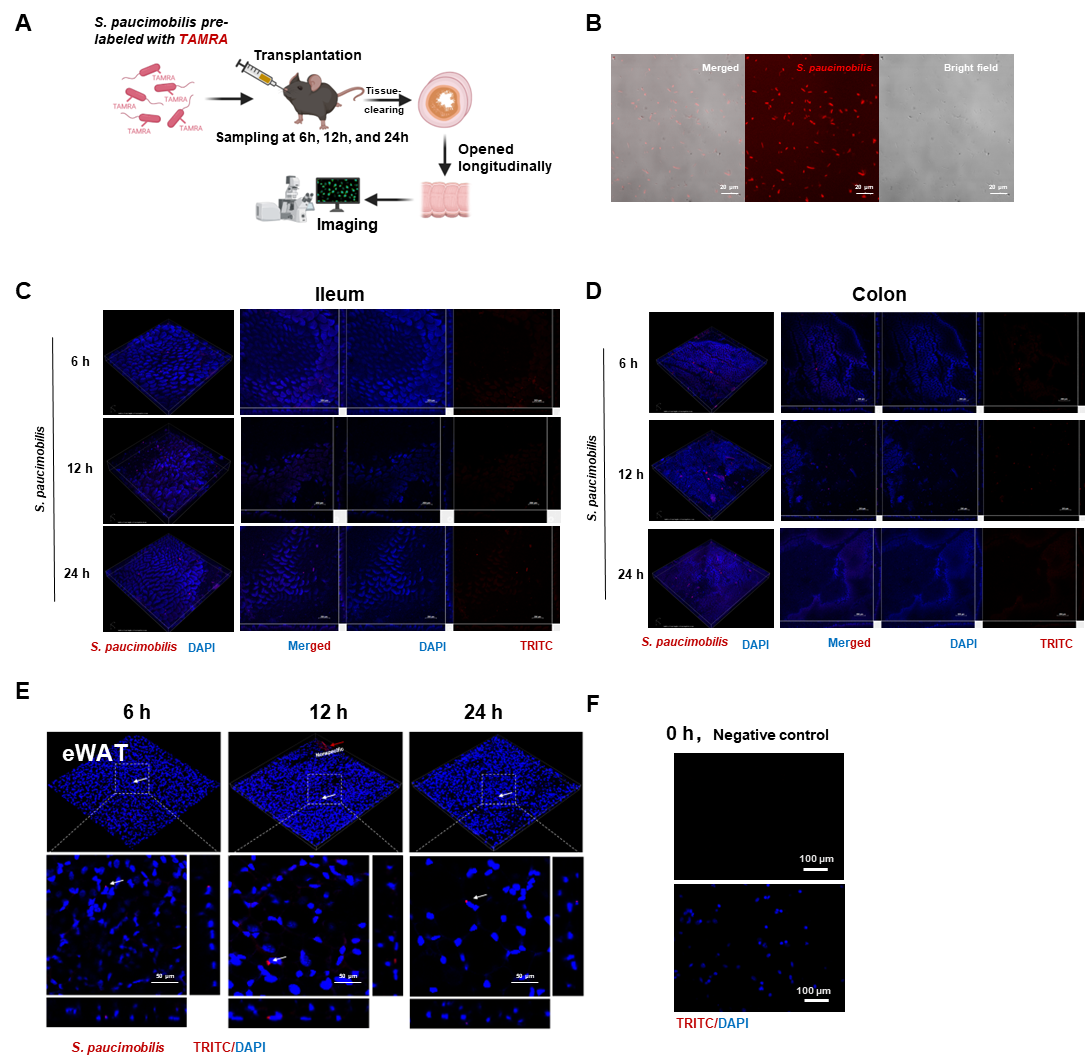
Figure S4. Tracking of *S. paucimobilis* in intestine and adipose tissue.**

(A) Schematic illustration of TAMRA labeled *S. paucimobilis* gavage experiment.

(B) Confocal images of *S. paucimobilis* labeled by TAMRA (red). Scale bar is 20μm.

(C-E) Representative images of ileum (C), colon (D), and eWAT (E) after TAMRA-labeled *S. paucimobilis* gavage. The *S. paucimobilis* and nuclei were stained with TAMRA probe (red) and DAPI (blue) respectively. Scale bar is 200 μm for C and D. Scale bar is 50μm for E.

(F) Adipose tissue before gavage was collected and stained as a negative control. Scale bar is 100 μm.

**
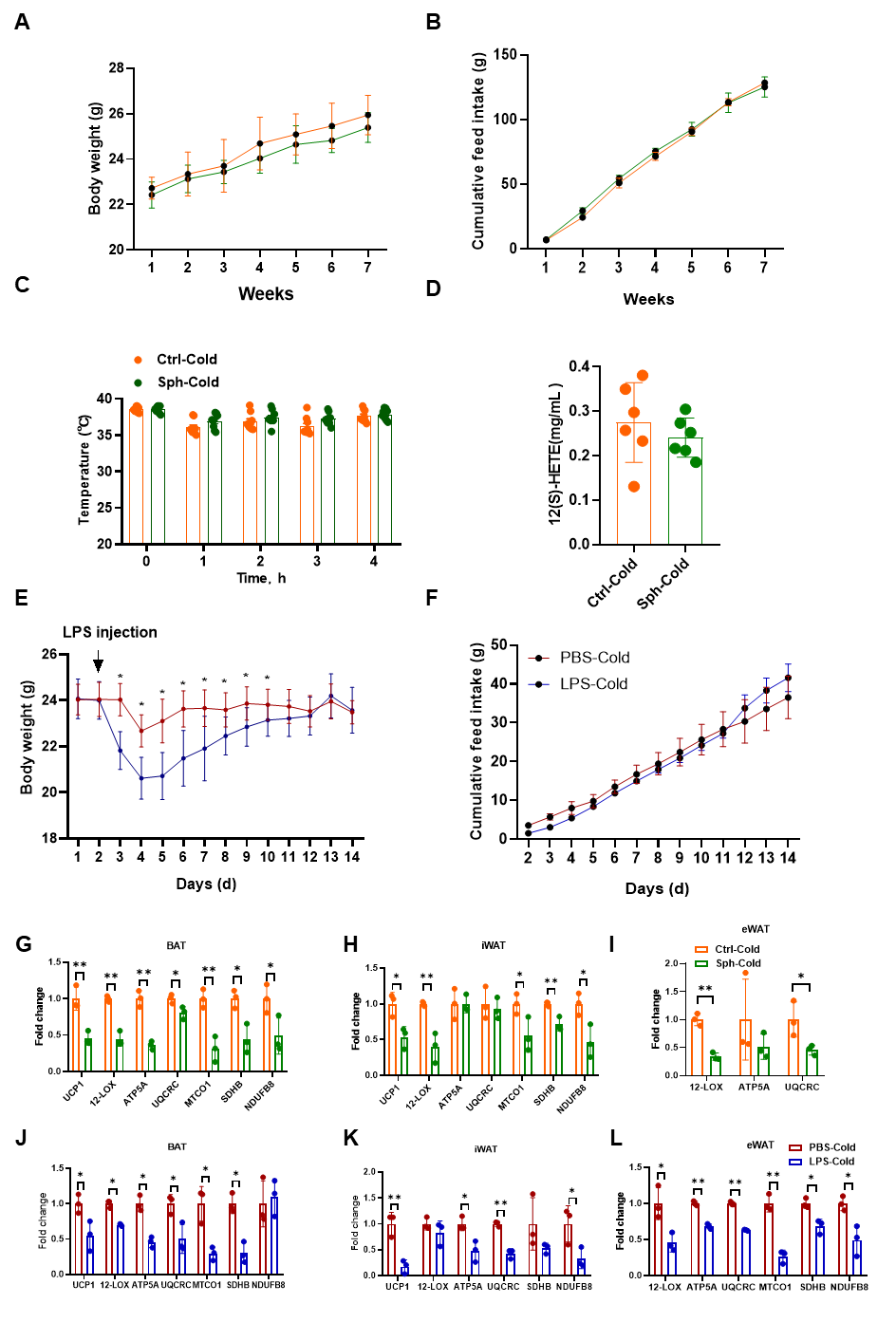
**

**Figure S5. *S. paucimobilis* inhibits adaptive thermogenesis.**

(A-B) Body weight (A) and cumulative food intake (B) in Ctrl-Cold and Sph-Cold groups, n =9.

(C) Core body temperature of *S. paucimobilis* -gavaged mice during cold exposure in 4 h, n = 9.

(D) The level of 12S-HETE in serum of *S. paucimobilis* -gavaged mice, n = 6.

(E-F) Body weight (E) and cumulative food intake (F) in PBS-Cold and LPS-Cold groups, n=14.

(G-I) Quantification of the western blot analysis of UCP1, 12-LOX, and OXPHOS f in BAT (G), iWAT (H), and eWAT (I) of Ctrl-Cold and Sph-Cold groups.

(J-L) Quantification of the western blot analysis of UCP1, 12-LOX, and OXPHOS in BAT (J), iWAT (K), and eWAT (L) of PBS-Cold and LPS-Cold groups. * *P* < 0.05; ** *P* < 0.01.


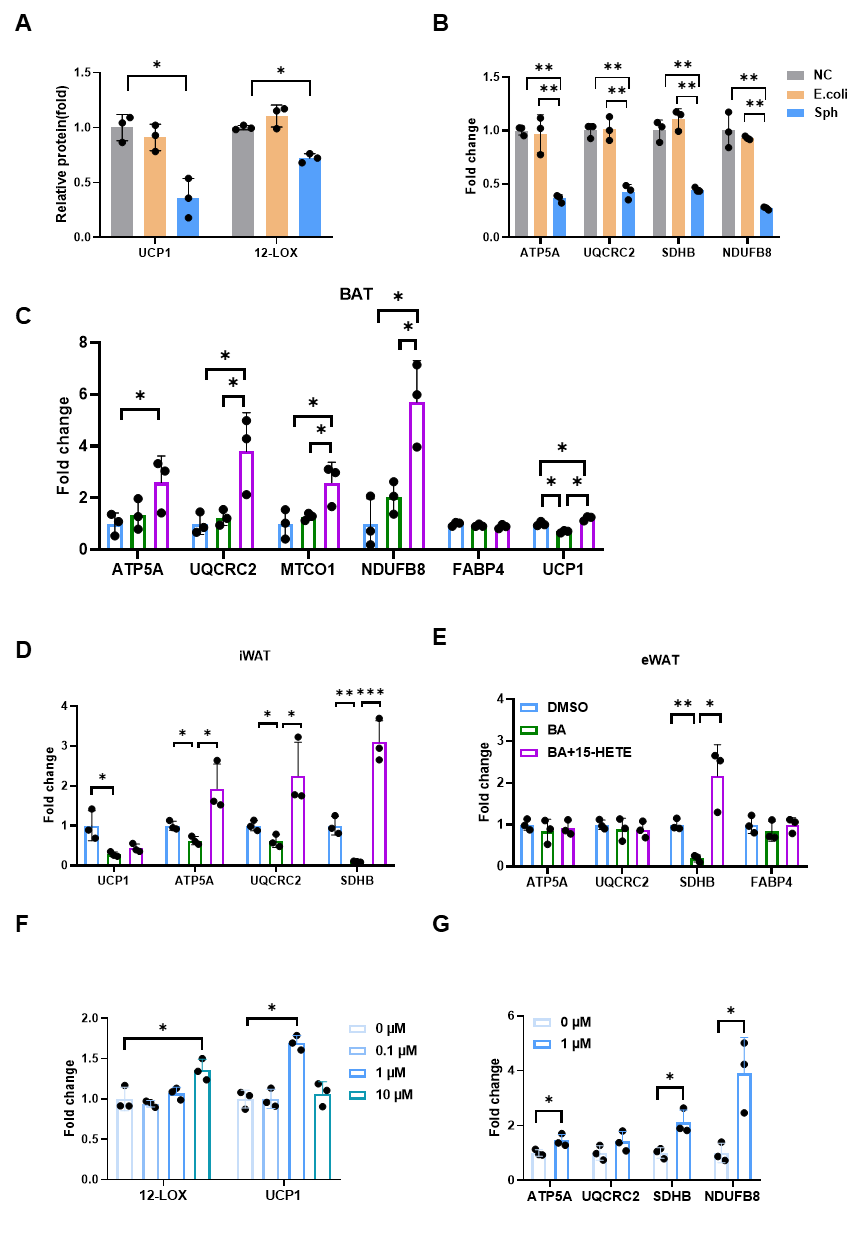


**Figure. S6. 15-HETE is an important metabolite for adaptive thermogenesis.**

(A-B) Quantification of the western blot analysis of UCP1, 12-LOX, and OXPHOS in primary adipocytes from eWAT treated by PBS, *E. coli*, or Sph.

(C-E) Quantification of the western blot analysis of UCP1, 12-LOX, and OXPHOS in BAT (C), iWAT (D), and eWAT (E) of DMSO, baicalein, and baicalein + 15-HETE groups.

(F-G) Quantification of the western blot analysis of UCP1, 12-LOX (F), and OXPHOS (G) in differentiated C3H10T1/2 cells incubated with 15-HETE. * *P* < 0.05; ** *P* < 0.01.

**
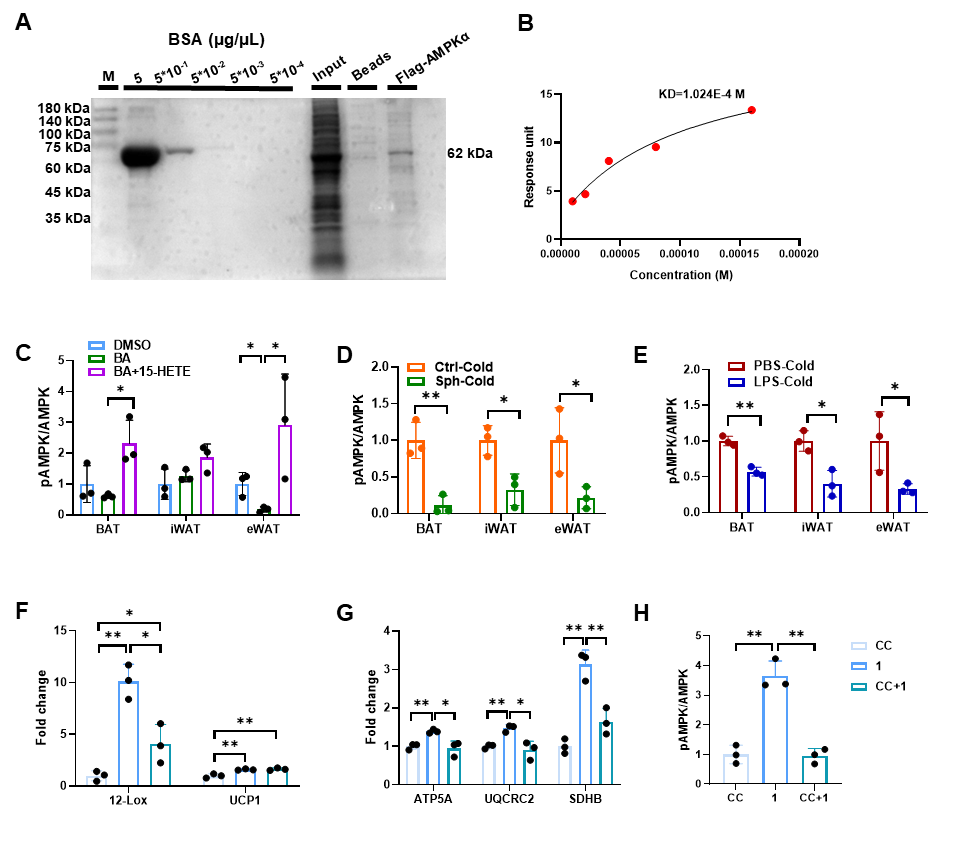
Figure S7. 15-HETE activates AMPK.**

(A) Purification of Flag-AMPKα.

(B) Concentration versus SPR sensor response between AMPKα and 15-HETE.

(C-E) Quantification of the western blot analysis of AMPK and pAMPK for adipose tissues in each group.

(F-H) Quantification of the western blot analysis of UCP1, 12-LOX (F), OXPHOS (G), AMPK and pAMPK (H) in 15-HETE- or CC- treated cells.


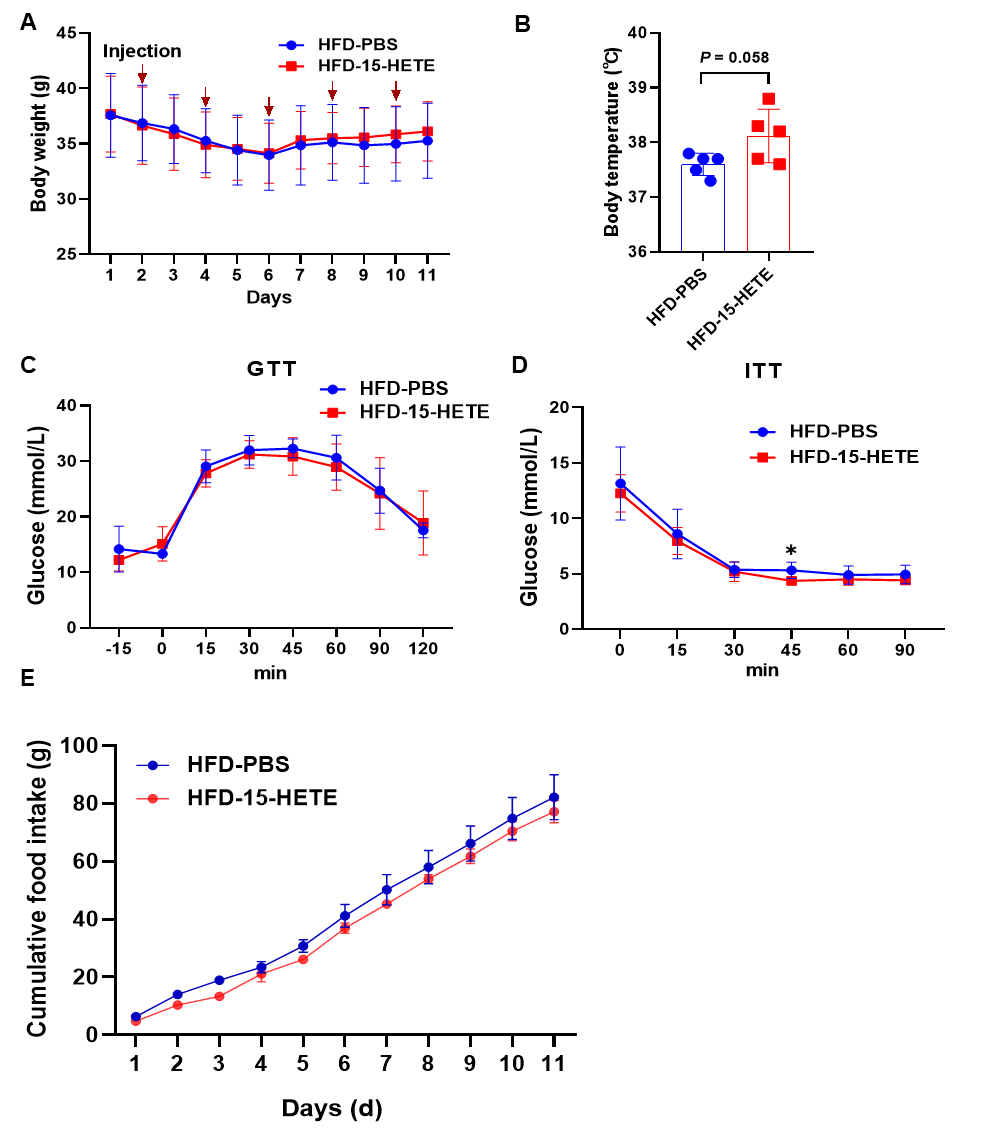


**Figure S8. 15-HETE increases body temperature of DIO mice.**

(A and B) Effect of 15-HETE on body weight (A) and body temperature (B) in DIO mice, n = 5-6.

(C and D) GTT (C) and ITT (D) for DIO mice injected with 15-HETE for 2 weeks, n = 4-5.

(E) Cumulative food intake in HFD-PBS and HFD-15-HETE-injected mice. * *P* < 0.05; ** *P* < 0.01.
